# Altered benzo[a]pyrene adduct formation in nucleosomes establishes distinct mutational patterns in lung cancer

**DOI:** 10.1016/j.jbc.2026.111291

**Published:** 2026-02-16

**Authors:** Benjamin Morledge-Hampton, Markus Lindberg, Erik Larsson, John J. Wyrick

**Affiliations:** 1 School of Molecular Biosciences, Washington State University, Pullman, Washington, USA; 2 Department of Medical Biochemistry and Cell Biology, Institute of Biomedicine, Sahlgrenska Academy at University of Gothenburg, Gothenburg, Sweden

**Keywords:** BPDE, lung cancer, NER, nucleosomes, transcription factors

## Abstract

Benzo[a]pyrene is a carcinogen in tobacco smoke that, when metabolized to benzo[a]pyrene diol epoxide (BPDE), induces mutagenic DNA lesions that promote the development of lung cancer. In lung cells, BPDE damages DNA packaged in nucleosomes, but the impact of nucleosomes on BPDE adduct formation is unclear. Here, we analyze genome-wide maps of BPDE adduct formation and repair in human cells. Our analysis indicates that BPDE adduct formation is suppressed in nucleosomes and enriched in adjacent linker DNA. Within nucleosomes, BPDE adduct formation is specifically elevated at minor-out rotational settings, where the minor groove of the DNA faces outward from the histone octamer. Structural analysis indicates that the solvent accessibility of the reactive exocyclic N2 amino group in guanine bases is elevated at minor-out rotational settings, potentially accounting for elevated BPDE damage at these locations. These damage patterns coincide with and can explain elevated somatic mutation rates in lung cancers at linker DNA and minor-out rotational settings in nucleosomes. While BPDE damage formation in nucleosomes strongly correlates with mutation patterns in lung cancers, the repair of these adducts does not. Analysis of damage patterns at CCCTC-binding factor and SP1 transcription factor binding sites indicates that BPDE damage formation is also suppressed by these DNA-bound proteins, and this damage modulation correlates with mutation patterns at these binding sites in lung cancers. These data indicate that altered BPDE adduct formation in chromatin can explain the distinct patterns of somatic mutations in lung cancers.

Lung cancer is one of the most commonly diagnosed and deadliest forms of cancer (1, 2). The disease is primarily caused by exposure to polycyclic aromatic hydrocarbons such as benzo[a]pyrene (3, 4, 5), which are produced by the incomplete combustion of organics, notably in cigarette smoke (6). Benzo[a]pyrene is metabolized by cellular enzymes to benzo[a]pyrene diol epoxide (BPDE), which can form adducts with DNA bases (usually guanine) that are mutagenic unless repaired (7), and a previous study has indicated that BPDE adduct formation is specifically elevated at mutation hotspots in the p53 gene in lung cancers (8). These bulky, helix-distorting adducts are detected and repaired by the nucleotide excision repair (NER) pathway (9, 10, 11). As a result, modulation of NER activity is implicated in BPDE-associated mutagenesis and the onset of lung cancer. Accordingly, recent work has reported that BPDE repair efficiency is primarily responsible for establishing mutation patterns in broad chromatin contexts such as early and late replicating regions (12). However, to what extent modulation of BPDE adduct formation contributes to genome-wide mutation patterns in lung cancers, particularly in discrete chromatin features like nucleosomes, is unclear.

Previous studies have shown that the packaging of DNA with histone proteins into nucleosomes significantly modulates somatic mutation rates in many cancers. In nearly a dozen different cancer types (*e.g.*, melanoma, esophageal and gastric cancers, *etc.*) (13, 14), somatic mutation rates are elevated near the center of strongly positioned nucleosomes and reduced in neighboring linker DNA. Since nucleosomes are known to inhibit both the base excision repair and NER pathways (14, 15, 16, 17), repair inhibition is thought to be responsible for elevated mutation rates in nucleosomal DNA in these cancer types. Genome-wide sequencing data from lung adenocarcinoma and lung squamous cell carcinoma tumors indicate that lung cancer is essentially the only cancer type with higher mutation rates in linker DNA relative to neighboring nucleosomes (14). However, the molecular mechanism responsible for elevated mutation rates in linker DNA is unknown.

Not only does the translational positioning of nucleosomes along DNA affect somatic mutation rates, but recent studies indicate that the precise rotational orientation of the DNA minor groove relative to the histone octamer also significantly modulates mutation rates in many cancers. Nearly 10 different types of cancers (*e.g.*, esophageal and gastric cancers, colorectal cancer, *etc.*) had elevated mutation rates at rotational positions where the DNA minor groove faces inward toward the histone octamer (dubbed “minor-in”) and reduced mutation rates where the minor groove faces outward (minor-out) (18). This results in an oscillating pattern of mutations in nucleosomal DNA, with a characteristic period of ∼10.2 bp, corresponding to the DNA helical repeat length (18). It has been suggested that these patterns likely originate from differential repair, as many repair pathways show elevated repair activity at minor-out positions, since such positions are more accessible to repair factors, and lower repair activity at inaccessible minor-in positions (13, 14, 15). However, a few cancer types (*e.g.*, melanoma and lung cancers) show the opposite pattern, as somatic mutations are specifically elevated at minor-out positions in nucleosomes and reduced at minor-in positions (13, 14). In the case of melanoma, this mutational pattern has been attributed to elevated UV damage formation at minor-out positions in nucleosomes (13, 14, 19, 20). Biochemical studies using reconstituted nucleosomes have reported that BPDE adduct formation is also modulated in nucleosomes *in vitro* (21), but to what extent nucleosomes modulate BPDE adduct formation in cells and whether this mechanism contributes to chromatin-associated patterns of lung cancer mutations remains unclear.

Transcription factors are another class of DNA-binding protein that have been shown to modulate UV-induced DNA damage, repair, and mutagenesis (22, 23, 24). For example, transcription factor binding sites (TFBSs) for a number of different transcription factor families, including ETS, SP1, and CCCTC-binding factor (CTCF), show elevated mutation rates in skin cancers such as melanoma, in many cases due to elevated UV damage formation and/or repair inhibition (24, 25, 26, 27). A previous report indicated that somatic mutations in lung adenocarcinomas and lung squamous cell carcinomas are also elevated at TFBSs (in aggregate) (22), and another study found both BPDE adduct depletion and enrichment across a broad selection of TFBSs (12). However, the relative contribution of BPDE damage and repair to lung cancer mutagenesis at TFBSs, especially for specific classes of transcription factors, is not understood.

In this work, we leverage existing maps of BPDE damage formation (12, 28) and repair activity (11) to investigate their contributions to nucleosomal patterns of mutagenesis in lung cancer (9). Our analysis indicates that BPDE adducts are suppressed in nucleosomes and specifically enriched in neighboring linker DNA, a pattern closely matching that observed for lung cancer mutations. Our analysis also indicates that BPDE adducts are enriched at minor-out positions in nucleosomes, which structural analysis indicates is due to increased solvent accessibility of the reactive exocyclic N2 amino group in guanine bases is elevated at minor-out rotational settings. These data imply that BPDE damage formation is modulated primarily by DNA accessibility, potentially explaining observed patterns of lung cancer mutagenesis in chromatin.

## Results

### BPDE adducts in cells are enriched in linker DNA

**Figure 1:**
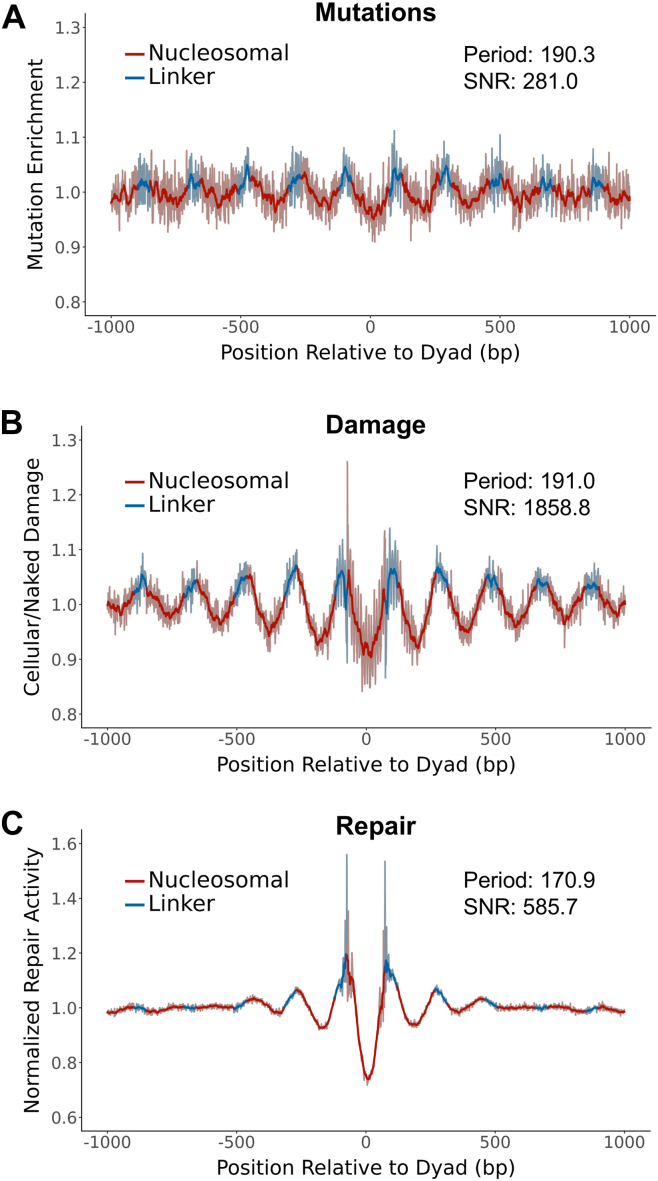
**Lung cancer mutations and BPDE damage formation are enriched in linker DNA.***A* and *C*, lung cancer mutation enrichment (*A*), BPDE damage enrichment (24h exposure) (*B*), and BPDE repair activity (*C*) with respect to translational nucleosome positioning based on a hybrid DNase-MNase nucleosome map. Mutation data (*A*) and repair data (*C*) are normalized by trinucleotide context. Damage data (*B*) are normalized by a naked DNA control. Dyad-relative positions are oriented 5′ to 3′ relative to the plus strand and are combined over both strands. *Darker lines* represent data smoothed in a sliding 11-bp window and are superimposed on the lighter-colored raw data. BPDE, benzo[a]pyrene diol epoxide.

Previous analysis has revealed that lung cancers show a unique pattern of somatic mutations in nucleosomes (14), but the molecular origin of this pattern is unclear. To address this question, we analyzed somatic mutations from whole genome sequencing of lung cancers (*i.e.*, LUAD and LUSC) from TCGA (29, 30). These data were analyzed alongside a published human nucleosome map derived from DNase-seq and MNase-seq data (31, 32, 33), and mutations were counted in a 2000-bp window centered on each strongly positioned nucleosome. As a control, we determined expected mutation frequencies at each nucleosomal position based on trinucleotide sequence context and calculated mutation enrichment by dividing the observed mutation counts by these expected counts. This analysis indicated that in lung cancers, somatic mutation enrichment peaked at linker positions and was lower in nucleosomes (Fig. 1*A*), consistent with previously published results (14). Lomb-Scargle analysis of the mutation enrichment data by mutperiod (34) revealed a significant periodicity of 190.3 bp (SNR [signal to noise ratio, a measure of the clarity of the periodic trend] = 281.0; Fig. 1*A*), corresponding to the length of DNA between each nucleosome dyad axis (*i.e.*, the nucleosome repeat length).

To investigate the impact of nucleosomes on BPDE formation, we analyzed a previously published map of BPDE adducts in human cells treated with 2 μM BPDE for 24 h (28). As a control, we compared the cellular BPDE damage map to a BPDE damage map in naked DNA from the same study, in which isolated human genomic DNA was exposed to BPDE under the same conditions. Analysis of these data indicated that BPDE adducts were enriched in linker DNA and suppressed in nucleosomes. This pattern had a highly significant period of 191.0 bp (SNR = 1285.8; Fig. 1*B*), which almost exactly matched the translational periodicity of the lung cancer mutation data (Fig. 1*A*).

This BPDE damage map was produced in repair-competent cells, meaning that the frequency of BPDE adducts after 24 h of BPDE exposure could be shaped not only by the rate of damage formation, but potentially by ongoing repair of these adducts. To ensure that the nucleosome patterns we observed were due primarily to damage formation, we repeated our analysis using a damage dataset produced after only 2 h of exposure to a higher dose of 25 μM BPDE (12). The results of this analysis closely matched those from the 24 h damage data, with higher damage formation rates in linker DNA relative to matched naked DNA controls (Fig. S1*A*). These findings indicate that the packaging of DNA into nucleosomes suppresses BPDE formation relative to adjacent linker DNA regions.

To examine the impact of nucleosomes on repair of BPDE damage by the NER pathway, we analyzed a previously published data set of BPDE tXR-seq reads (11). The likely position of the repaired guanine lesion was inferred for each tXR-seq read length (see Experimental procedures), and the resulting repair activity data were normalized by the genome-wide frequency of tXR-seq lesions in each trinucleotide sequence context. Analysis of BPDE tXR-seq data indicated that repair of BPDE adducts by the NER pathway is elevated in linker DNA and suppressed in nucleosomes (Fig. 1*C*). More detailed analysis also indicates that the translational period for the tXR-seq repair data is distinct from those observed in the damage and mutation data (period = 170.9 bp; SNR = 585.7; Fig. 1*C*). Taken together, these findings indicate that BPDE adduct formation, not NER activity, is highly correlated with somatic mutation rates in nucleosomes, with both elevated damage and mutations in accessible linker regions.

These results were obtained using a previously published hybrid DNase-seq and MNase-seq nucleosome map (31, 32, 33), which is enriched for more strongly positioned nucleosomes at the cost of overall genome coverage (1,011,966 nucleosomes total). We also analyzed the data using a less stringent MNase-only map (14, 32, 35) containing over ten times the number of nucleosomes (11,901,840 nucleosomes). These results were comparable, as both BPDE adduct formation and lung cancer mutations were significantly elevated in linker DNA (Fig. S2). An exception is the tXR-seq repair data, which displayed a more sporadic periodicity at positions closest to the central nucleosome and a much higher period (period = 226.5 bp; SNR = 152.4; Fig. S2*C*), which still did not match the period of the lung cancer mutation data for either nucleosome map. These differences in NER activity could reflect the difference in the strength of nucleosome positioning between the two maps, since the MNase-only map contains more weakly positioned nucleosomes, which may be more permissive to repair.

### BPDE adduct formation is elevated at minor-out rotational settings in nucleosomes

**Figure 2:**
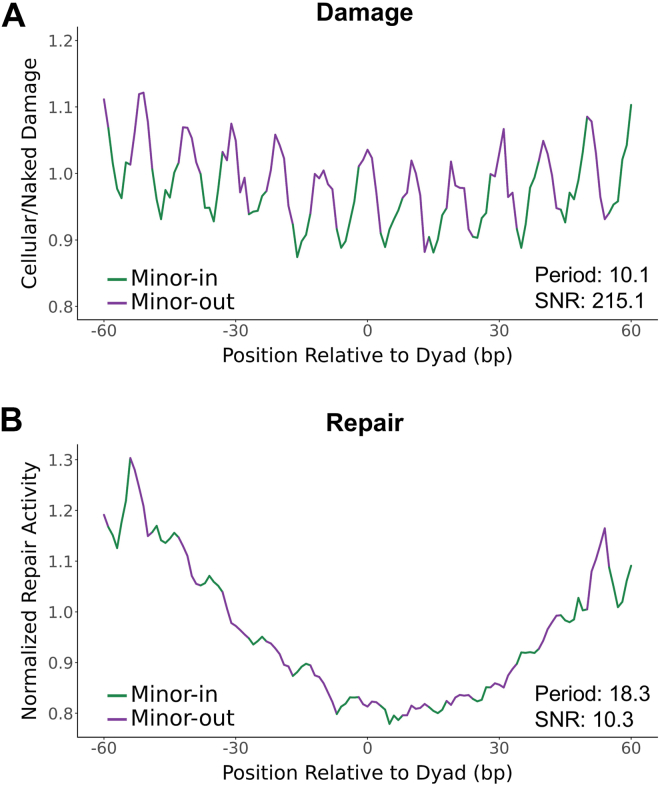
**BPDE adduct formation is increased at minor-out positions.***A and B*, BPDE damage enrichment (24h exposure) (*A*) and repair activity (*B*) with respect to rotational nucleosome positioning based on a hybrid DNase-MNase nucleosome map. Damage data (*A*) are normalized by a naked DNA control. Repair data are normalized by trinucleotide context. Dyad-relative positions are oriented 5′ to 3′ relative to the plus strand and are combined over both strands. BPDE, benzo[a]pyrene diol epoxide.

Given that the enrichment of BPDE lesion formation can explain elevated lung cancer mutations in nucleosome-free linker DNA, we wondered if modulation of BPDE lesions in nucleosomal DNA may also be responsible for elevated mutation rates at minor-out rotational settings. Detailed analysis of BPDE damage formation within the nucleosome core (*i.e.*, positions −60 to +60 bp from the central nucleosome dyad axis) following 24h of exposure revealed that BPDE damage rates were consistently higher at minor-out positions and lower at minor-in positions (period = 10.1 bp; SNR = 215.1; Fig. 2*A*), and data from the 2h damage map also displayed this pattern (Fig. S1*B*). In contrast, repair data were largely unaffected by the orientation of the minor groove (period = 18.3 bp; SNR = 10.3; Fig. 2*B*). These results were obtained using the hybrid DNase-seq and MNase-seq based nucleosome map; similar results were obtained when analyzing BPDE damage formation in the larger MNase-only map (Fig. S3). A previous study (14) indicated that lung cancer mutations are also enriched at minor-out positions. We observed the same trend in our analysis, with a relatively weak signal in the lower-coverage hybrid nucleosome map (period = 10.1 bp; SNR = 8.4; Fig. S4*A*), but a much clearer periodic pattern of somatic mutations in the larger MNase-only map (period = 10.2 bp; SNR = 42.9; Fig. S4*B*).

**Figure 3:**
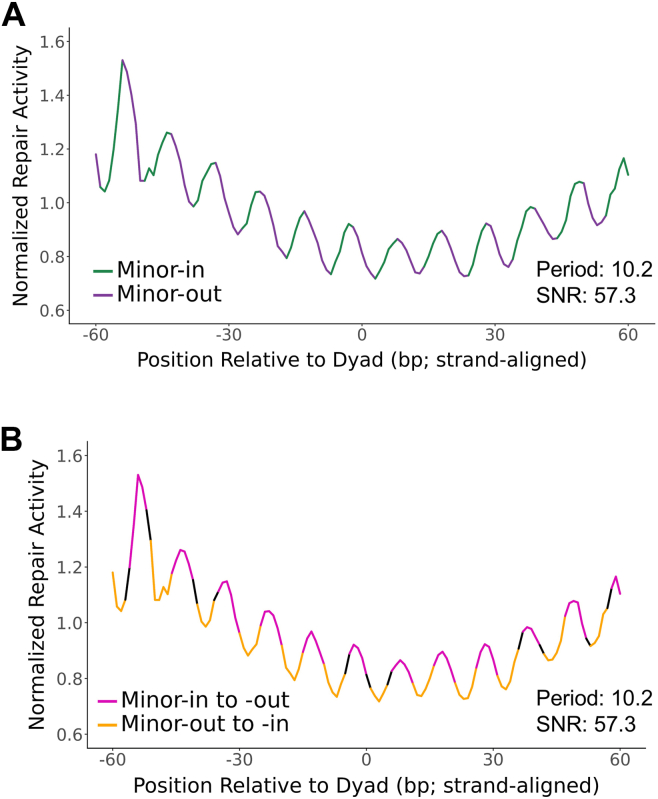
**BPDE repair activity displays a strand-specific rotational periodicity.***A* and *B*, BPDE repair activity with respect to rotational nucleosome positioning based on a hybrid DNase-MNase nucleosome map, highlighting either the position of the minor groove (*A*) or transitions between these positions (*B*). Data are normalized to trinucleotide context. Dyad-relative positions are oriented 5′ to 3′ for both strands. (*i.e.*, minus-strand positions were inverted about the dyad axis so that both strands are aligned in the same direction.). BPDE, benzo[a]pyrene diol epoxide.

Curiously, while BPDE repair activity had no clear rotational periodicity with respect to the orientation of the minor groove (Fig. 2*B*), a distinct rotational periodicity was revealed by orienting the data from both DNA strands in the 5′ to 3′ direction (Fig. 3). Normally, aligning the two DNA strands in a 5′ to 3′ orientation is not necessary to observe the effects of rotational setting on damage or repair, because the orientation of the minor-groove is symmetrical about the dyad axis (36). However, the observed rotational periodicity does not appear to be directly influenced by minor-in or minor-out positions (Fig. 3*A*) and is instead most pronounced at the transitions between these states (Fig. 3*B*). These results were clearest for the hybrid nucleosome map (period = 10.2 bp; SNR = 57.3; Fig. 3) but were also observable in the MNase-only map (period = 10.1 bp; SNR = 31.5; Fig. S5). The transition between minor-in to minor-out reflects positions where the DNA backbone is facing inward toward the histone octamer, which may be more permissive for damage recognition and repair (see Discussion). In summary, these results show that patterns of BPDE damage formation (but not repair activity) in nucleosomes are associated with and can potentially explain the mutational periodicities observed in lung cancers.

### Structural analysis suggests a molecular mechanism for elevated BPDE adduct formation at minor-out positions in nucleosomes

**Figure 4:**
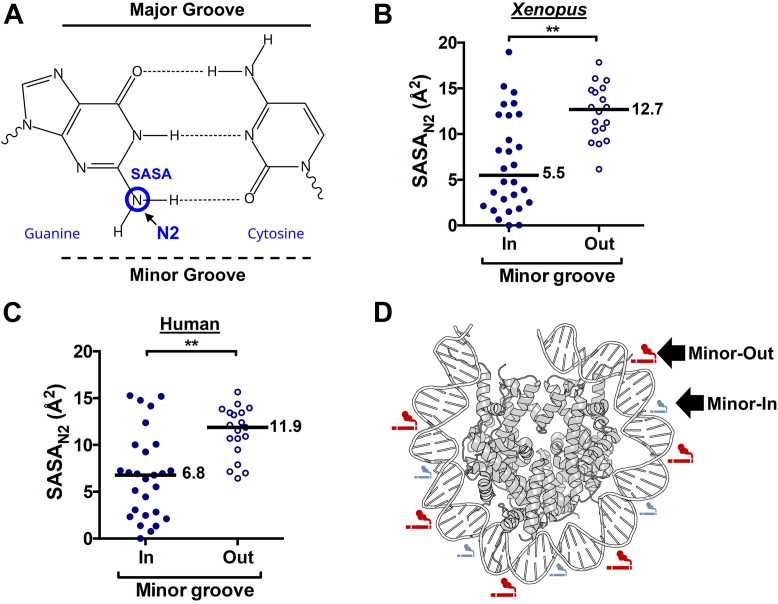
**Solvent accessibility of the reactive N2 position of guanine is modulated by nucleosomal rotational positioning.***A*, schematic of a guanine-cytosine base pair, highlighting the position of the BPDE-reactive exocyclic N2 amino group of guanine, which is located on the minor-groove side of the base pair. Solvent accessible surface area was calculated for this atom at guanine bases in nucleosome structures. *B*, solvent accessible surface area of the N2 atom in guanine nucleotides (SASA_N2_) for minor-in and minor-out positions in the *Xenopus* nucleosome structure (1KX5). The median SASA_N2_ for minor-in and minor-out positions are indicated. ∗∗*p* < 0.001 based on Mann-Whitney test. *C*, same as *panelB*, except for the human nucleosome structure (2CV5). *D*, model of the human nucleosome structure, indicating that BPDE damage formation is elevated at minor-out positions (indicated by *red cigarette smoke symbols*) and suppressed at minor-in positions (indicated by *smaller blue symbols*) in nucleosomes. Only one of the DNA gyres is shown. The nucleosome structure (PDB ID: 2CV5) was generated using pymol. BPDE, benzo[a]pyrene diol epoxide.

BPDE primarily forms covalent adducts at the exocyclic N2 amino group in guanine nucleotides (37, 38). Since the N2 position is located on the minor-groove side of the DNA double helix, we wondered whether differences in the solvent-accessible surface area of the N2 atom (*i.e.*, SASA_N2_) might explain the differential reactivity of guanine bases at minor-in *versus* minor-out rotational settings in the nucleosome (Fig. 4*A*).

To test this hypothesis, we analyzed the SASA_N2_ of guanine bases in a high-resolution X-ray structure of the *Xenopus* nucleosome. While there is considerable variability in the SASA_N2_ of guanine bases in the nucleosome, our data indicated that the median solvent accessibility of guanine bases is ∼2-fold higher at minor-out rotational settings in nucleosomes compared to minor-in positions (Fig. 4*B*, *p* < 0.001). Similar analysis of the human nucleosome structure produced comparable results (Fig. 4*C*, *p* < 0.001). Furthermore, we also calculated the SASA_N2_ for the *Xenopus* nucleosome with an alternative DNA sequence (601 (39)) to confirm these results. This analysis also produced a significant difference in SASA_N2_ favoring minor-out rotational settings (Fig. S6). These findings suggest that differences in solvent accessibility of the guanine N2 amino groove due to the orientation of the DNA minor groove relative to the histone octamer can potentially explain the observed patterns of BPDE adduct formation in nucleosomes (Fig. 4*D*).

### BPDE damage and repair are altered at transcription factor binding sites

A previous study observed higher rates of lung cancer mutations associated with TFBSs (22), but the mechanism involved was unclear. We hypothesized that transcription factors bound to DNA may also impact BPDE damage formation and repair. To test this hypothesis, we analyzed the same BPDE damage (12, 28) and repair (11) maps relative to 30,853 CTCF and 19,012 SP1 binding sites identified by the ENCODE project (40, 41). We tested these sites because they are among the most abundant TFBS classes in the human genome, and previous studies have indicated that they may modulate UV damage formation and/or repair (22, 26, 27).

**Figure 5:**
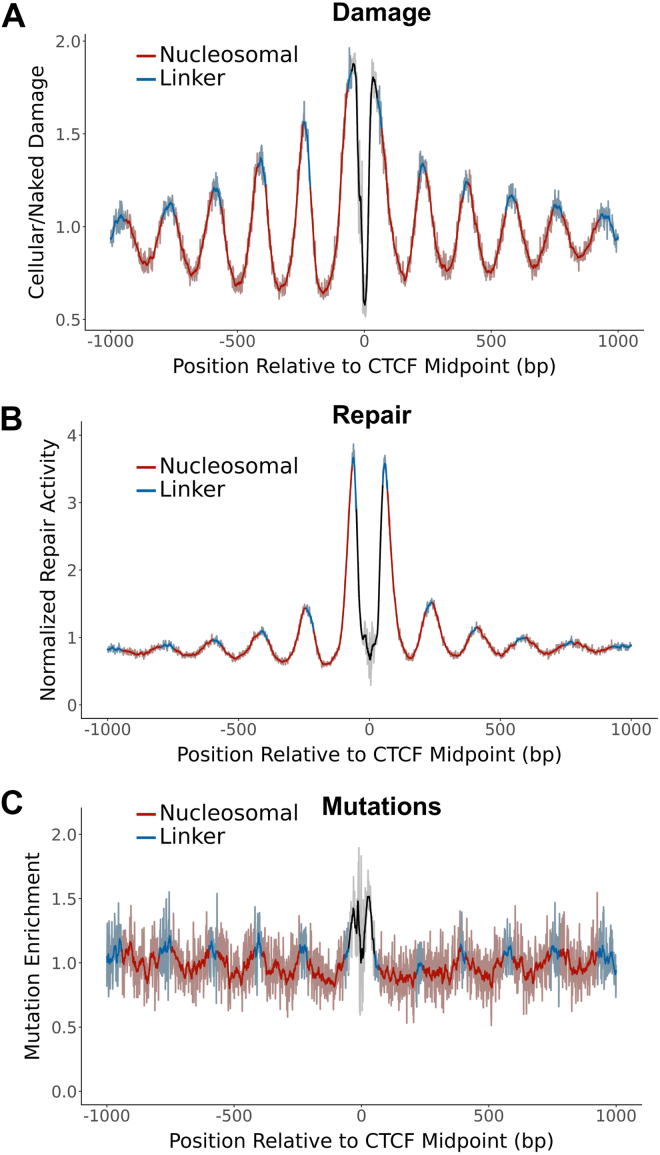
**CTCF binding sites and surrounding nucleosomes modulate BPDE damage, BPDE repair, and lung cancer mutagenesis.***A* and *C*, BPDE damage enrichment (2h exposure) (*A*), BPDE repair activity (*B*), and lung cancer mutation enrichment (*C*) surrounding CTCF binding sites. Damage data (*A*) are normalized by a naked DNA control. Repair (*B*), and mutation (*C*) data are normalized by trinucleotide context. Positions are oriented 5′ to 3′ relative to the strand containing the CTCF binding motif and are combined over both strands. Darker lines represent data smoothed in a sliding 11-bp window and are superimposed on the lighter-colored raw data. Nucleosomal and linker regions were determined by calling peaks of nucleosome dyads from a hybrid DNase-MNase nucleosome map, omitting regions within 100 bp of the CTCF binding sites (Fig. S8). BPDE, benzo[a]pyrene diol epoxide.

To characterize the effect of CTCF binding on BPDE formation, we analyzed BPDE adduct enrichment in BPDE-exposed cells relative to the naked DNA control at 30,853 CTCF binding sites. Because preliminary analysis indicated that very high NER activity in nucleosome-free regions immediately flanking CTCF binding sites may suppress BPDE damage levels observed in the 24h damage map (Fig. S7), we only utilized the high-dose 2h damage map for this analysis. Our results indicated that BPDE damage enrichment was suppressed at CTCF binding sites but elevated in nucleosome-free regions immediately flanking each binding site (Fig. 5*A*). Further from the binding site, BPDE damage enrichment displayed a clear periodicity that roughly matched the expected repeat length for positioned nucleosomes (Fig. 5*A*). Analysis of nucleosome dyad positions neighboring CTCF binding sites (Fig. S8) indicated that BPDE damage enrichment peaked at linker regions in the ordered nucleosome arrays surrounding CTCF binding sites (Fig. 5*A*), consistent with our previous results (see Fig. 1).

Similar analysis of the BPDE tXR-seq repair data indicated that repair activity is also relatively low at the CTCF binding site and elevated at positions immediately flanking it (Fig. 5*B*). These results are consistent with prior studies indicating that CTCF binding inhibits NER of helix-distorting DNA lesions (22, 26, 27). Repair activity was also elevated in linker DNA regions associated with the ordered nucleosome arrays flanking CTCF binding sites (Fig. 5*B*), consistent with our prior analysis. Additionally, lung cancer mutations around CTCF binding sites displayed the same nucleosome pattern, with elevated mutation enrichment in linker DNA and lower enrichment in nucleosomes (Fig. 5*C*).

**Figure 6:**
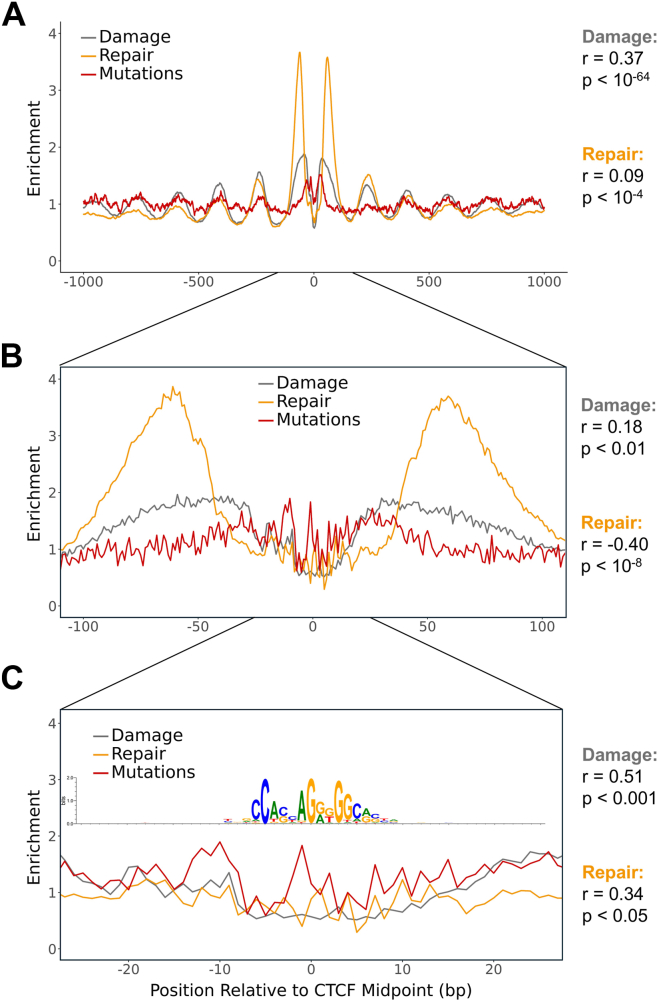
**The correlation between lung cancer mutagenesis and BPDE damage or repair varies with proximity to CTCF binding sites.***A* and *C*, BPDE damage enrichment (2h exposure), BPDE repair activity, and lung cancer mutation enrichment in a 2000-bp window (*A*), 200-bp window (*B*), or 50-bp window (*C*) around CTCF binding sites. For the 2000-bp window (*A*), data are smoothed in a sliding 11-bp window. For the 200-bp window (*B*) and 50-bp window (*C*), data are not smoothed. Damage data are normalized by a naked DNA control, while repair and mutation data are normalized by trinucleotide context. Positions are oriented 5′ to 3′ relative to the strand containing the CTCF binding motif and are combined over both strands. The sequence logo (*C*) represents the relative information content of positions surrounding CTCF binding midpoints. BPDE, benzo[a]pyrene diol epoxide.

To better understand the relationship between lung cancer mutations, BPDE damage, and BPDE repair at CTCF binding sites, we measured the Pearson correlation between mutation enrichment and damage formation as well as the correlation between mutation enrichment and repair activity. When this analysis was performed on the entire region of interest (1000 bp of DNA on either side of CTCF binding midpoints), the results indicated that mutation enrichment correlated more strongly with damage (r = 0.37; *p* < 10^−64^) than repair (r = 0.09; *p* < 10^−4^; Fig. 6*A*). Given the abundance of strongly positioned nucleosomes within 1000 bp of CTCF binding sites (Fig. S8), these results are most likely attributable to damage and repair patterns in flanking nucleosomes, rather than the CTCF binding sites themselves, and again indicate that elevated damage formation in linker DNA can potentially explain this mutation pattern in lung cancers.

To investigate the impact of the chromatin environment immediately surrounding the CTCF binding site (*i.e.*, independent of the surrounding nucleosomes) on mutagenesis, we repeated the correlation analysis in 200-bp and 50-bp windows around the binding midpoints. In the 200-bp window, which encompassed the prominent damage and repair peaks flanking the binding site, mutation enrichment correlated weakly with damage (r = 0.18; *p* < 0.01) but showed a strong negative correlation with repair activity (r = −0.40; *p* < 10^−8^; Fig. 6*B*). In the 50-bp window, where both damage and repair enrichment were relatively low, mutation enrichment was strongly correlated to damage (r = 0.51; *p* < 0.001) and displayed an unexpected positive correlation with repair activity (r = 0.34; *p* < 0.05; Fig. 6*C*).

Given the inherent link between DNA damage and repair (*i.e.*, areas with abundant damage tend to also have higher repair rates due to increased substrate availability), we repeated these correlation analyses with BPDE repair data normalized by BPDE damage instead of trinucleotide context. For each analyzed region, the correlation coefficient for mutation *versus* repair enrichment became more negative, but the same relative trends persisted, with a stronger, positive correlation between mutation and damage in the 2000-bp and 50-bp windows and a stronger, negative correlation between mutation and repair in the 200-bp window (Fig. S9). Taken together, these findings indicate that BPDE damage enrichment is significantly correlated with somatic mutations in lung cancers, both in the immediate vicinity of CTCF binding sites (*i.e.*, 50-bp window) and in flanking nucleosome arrays. In contrast, very high repair activity in nucleosome-free DNase I hypersensitivity regions immediately flanking CTCF binding sites (Figs. 6*B* and S9) suppresses mutagenesis in these regions, despite high damage formation, consistent with a previous report (12).

**Figure 7:**
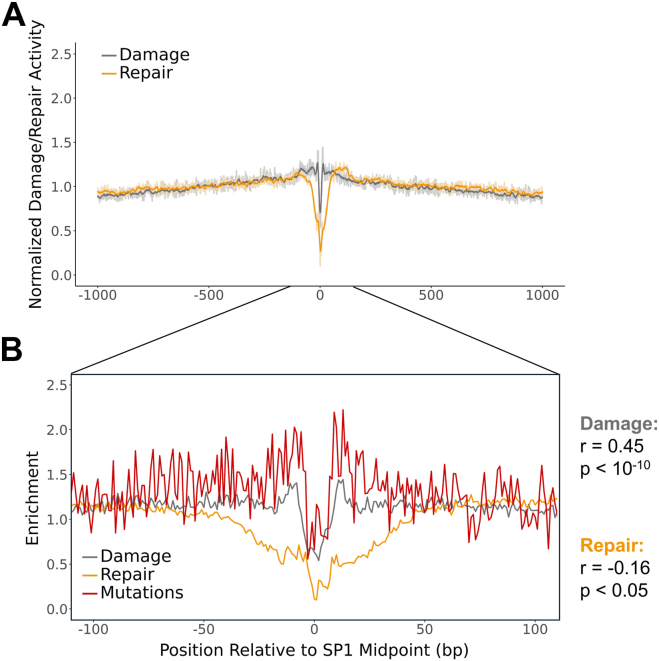
**BPDE damage, BPDE repair, and lung cancer mutagenesis are modulated at SP1 binding sites.***A* and *B*, BPDE damage enrichment (2h exposure) and repair activity in a 2000-bp window (*A*) or 200-bp window including lung cancer mutation enrichment (*B*) around SP1 binding sites. For the 2000-bp window (*A*), darker lines represent data smoothed in a sliding 11-bp window and are superimposed on the lighter-colored raw data. All data in the 200-bp window (*B*) are not smoothed. Damage data are normalized by a naked DNA control, while repair and mutation data are normalized by trinucleotide context. Positions are oriented 5′ to 3′ relative to the strand containing the SP1 binding motif and are combined over both strands. BPDE, benzo[a]pyrene diol epoxide.

Unlike CTCF binding sites, SP1 binding sites do not establish a nucleosome periodicity in the flanking DNA (Fig. S10). Accordingly, most of the DNA within 1000 bp of these TFBSs displays little variation in BPDE damage enrichment or repair activity (Fig. 7*A*). However, near the binding midpoint, there was a decrease in BPDE damage and repair activity. Decreased damage enrichment and repair activity near SP1 binding sites coincided with lower mutation enrichment in lung cancers. Correlation analysis in the same 100-bp region surrounding SP1 binding site midpoints revealed a strong, positive correlation between mutations and damage (r = 0.45; *p* < 10^-10^) and a weak, negative correlation between mutations and repair (r = −0.16; *p* < 0.05; Fig. 7*B*), even after normalizing repair activity by damage levels instead of trinucleotide context (r = −0.24; *p* < 0.001; Fig. S11). Altogether, these results indicate that modulation of BPDE adduct formation correlates with and can potentially explain mutation patterns, not only in and adjacent to nucleosomes, but at transcription factor binding sites as well.

### BPDE damage formation and mutagenesis are modulated at active transcription start sites

**Figure 8:**
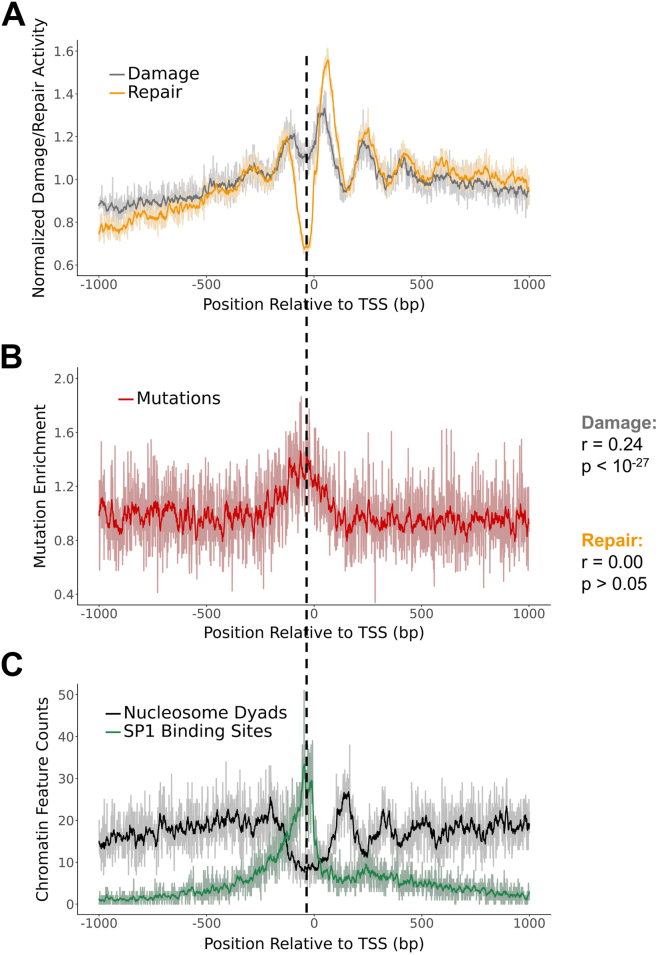
**BPDE damage enrichment correlates with lung cancer mutagenesis at transcription start sites.***A* and *C*, BPDE damage enrichment (2h exposure) *versus* repair activity (*A*), lung cancer mutation enrichment (*B*), and nucleosome and SP1 localization (C) surrounding transcription start sites (TSS). Damage data are normalized by a naked DNA control, while repair and mutation data are normalized by trinucleotide context. Positions are oriented 5′ to 3′ relative to the transcribed strand and are combined over both strands. Darker lines represent data smoothed in a sliding 11-bp window and are superimposed on the lighter-colored raw data. The dotted line indicates the center of the region of low nucleosome occupancy and frequent SP1 binding across all three plots. BPDE, benzo[a]pyrene diol epoxide.

Bindings sites for SP1 and other transcription factors are enriched in the core promoter region immediately adjacent to transcription start sites (TSSs) in human genes (42) while nucleosomes tend to be depleted from these same regions (43). Hence, we wondered how chromatin organization surrounding the TSS affects BPDE damage formation and repair. To address this question, we analyzed BPDE damage patterns surrounding ∼20,000 protein coding TSSs identified using the GENCODE hg19 genome annotations (44). This analysis showed a broad peak of BPDE damage formation surrounding the TSS (Fig. 8*A*), consistent with previous reports (12, 28). This damage peak roughly colocalized with a peak in lung cancer mutations (Fig. 8*B*). Moreover, in the 2 kb window surrounding the TSS, BPDE damage significantly correlated with mutation enrichment (r = 0.24; *p* < 10^-27^). Additionally, this link between damage enrichment and lung cancer mutagenesis was absent in the TSSs of the bottom quartile of expressed genes (see Methods) but still pronounced among TSSs in the top quartile (Fig. S12). Analysis of positioned nucleosomes flanking the TSS indicated that peaks of BPDE formation were associated with the nucleosome depleted region immediately surrounding the TSS as well as downstream linker regions (Fig. 8*C*).

Similar analysis of the BPDE tXR-seq data revealed peaks of NER activity in accessible linker regions but much lower repair activity in the center of the nucleosome depleted region surrounding the TSS (Fig. 8*A*). Unlike BPDE damage, NER activity did not significantly correlate with mutation enrichment in the 2 kb window surrounding the TSS (r = 0.00; *p* > 0.05). Analysis of the distribution of SP1 binding sites surrounding the TSSs revealed that repair activity was lowest where SP1 binding sites were most frequent (Fig. 8*C*). Taken together, these findings suggest that the unique chromatin environment surrounding active TSSs modulates BPDE damage formation and repair, with damage formation and lung cancer mutations peaking in the nucleosome depleted region surrounding the TSS.

## Discussion

**Figure 9:**
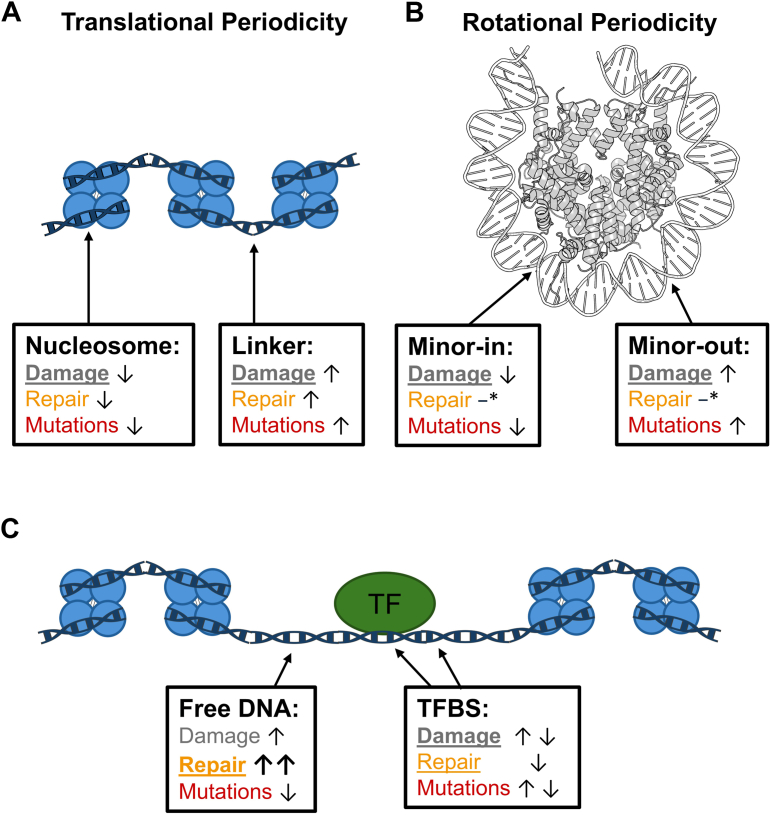
**Nucleosomes, transcription factors, and free-DNA correlate with changes in BPDE damage, BPDE repair, and lung cancer mutagenesis.***A*–*C*, relative BPDE damage enrichment, BPDE repair activity, and lung cancer mutation enrichment in nucleosomes (*A* and *B*) and transcription factor binding sites (*C*). Features (damage or repair) which correlate strongly with mutation enrichment in each context are underlined. Arrows pointing up or down represent overall increased or decreased enrichment, respectively, while more complex interactions are represented by opposing *arrows* (*C*). ∗Repair within nucleosomes (*B*) does not appear to be modulated with respect to the minor groove but instead correlates with the location of the phosphate backbone relative to the nucleosome dyad in single-stranded DNA (Fig. S5). BPDE, benzo[a]pyrene diol epoxide. Image of nucleosome structure was generated using pymol from PDB ID: 2CV5.

Somatic mutations in lung cancers are elevated in linker DNA and suppressed in nucleosomes, particularly at minor-in positions, a pattern opposite from nearly all other cancer types (14). BPDE lesions and related adducts are a major source of somatic mutations in lung cancer (3, 4, 5), but whether modulation of BPDE damage formation or repair in nucleosomes can potentially explain these mutation patterns was previously unclear. By leveraging genome-wide maps of BPDE damage (12, 28) and repair (11), we show that BPDE adducts form preferentially at accessible linker DNA adjacent to nucleosomes and at minor-out positions within nucleosomal DNA. Elevated damage formation in linker DNA and minor-out positions coincides with, and can potentially explain, higher mutation rates in these locations in lung cancer (Fig. 9, *A* and *B*). In contrast, our results indicate that repair activity shows less consistent patterns with respect to nucleosome positioning and the orientation of the DNA minor-groove and cannot explain the observed patterns of lung cancer mutations in nucleosomes (Fig. 9, *A* and *B*). We show that BPDE damage formation is also modulated by other DNA-binding proteins, such as the transcription factors CTCF and SP1, with lower damage occurring within their binding sites, and in the case of CTCF, higher damage in nucleosome-free flanking regions (Fig. 9*C*). These results indicate that chromatin and other DNA-bound proteins alter the susceptibility of DNA to BPDE damage, and that this damage proclivity plays a key role in establishing patterns of mutagenesis in lung cancer.

Our analysis indicates that BPDE damage levels are elevated in linker DNA and coincide with increased somatic mutation enrichment in human lung cancers (14). BPDE repair activity from tXR-seq experiments (11) is often elevated in these regions, indicating that repair activity is generally inconsistent with the observed mutation patterns. Our finding that BPDE damage formation is suppressed in nucleosomal DNA across the human genome is consistent with prior biochemical studies, which indicated that nucleosome assembly suppresses the levels of BPDE-adduct formation *in vitro*.

However, these prior biochemical studies did not observe a clear effect of rotational positioning on BPDE adduct formation (21, 45, 46). In contrast, our analysis of genome-wide damage maps suggests that BPDE lesions are elevated at minor-out positions in nucleosomes, which can potentially explain why lung cancer mutations are also elevated at these positions. This discrepancy is likely due to the use of a single nucleosome positioning sequence (*e.g.*, the 5S ribosomal DNA sequence) in prior biochemical studies, since the impact of DNA sequence context, which is known to significantly modulate BPDE formation (21, 46), can potentially overwhelm the impact of rotational setting when only a single positioning sequence is analyzed. In contrast, our analysis mitigates the effects of DNA sequence context by analyzing damage formation in nucleosomes throughout the genome and by comparing the cellular damage levels to a naked DNA control.

A key question is what molecular mechanism(s) are responsible for the observed patterns of BPDE damage formation in chromatin. It has been suggested that BPDE non-covalently interacts with DNA prior to reacting with the exocyclic N2 amino group of guanine to form a covalent adduct (21, 46). Based on this model, it is likely that the wrapping of DNA around the histone octamer sterically hinders such interactions with BPDE, thereby reducing BPDE adduct formation in nucleosomes and other protein-bound DNA regions. Within nucleosomes, our structural analysis suggests that the reactive N2 atom in guanine bases has elevated solvent accessibility at minor-out positions and reduced solvent accessibility at minor-in positions, a consequence of the N2 atom being on the minor-groove side of the guanine-cytosine base pair. This feature of the nucleosome structure can potentially explain the observed rotational periodicity of BPDE adduct formation and suggests a molecular mechanism driving elevated somatic mutation rates at minor-out positions in lung cancers.

While BPDE repair activity did not show a consistent rotational pattern in nucleosomes when analyzed as double-stranded DNA, we did observe a strand-specific rotational periodicity in repair activity. Our analysis indicated that NER activity is highest when the lesion is located at the transition from minor-in to minor-out regions, near where the DNA backbone of one strand is oriented toward the histone octamer (*i.e.*, backbone-in) (47). Previous studies have shown that BPDE adducts can affect the stability of nucleosomes (48, 49), particularly for BPDE-adducts in which the DNA backbone is oriented toward the histone octamer (48). It is possible that adduct-induced changes in nucleosome stability could potentially explain why repair activity is elevated for backbone-in BPDE adducts. Our analysis also indicates that NER activity in BPDE-treated cells is suppressed in nucleosomes relative to linker DNA and other nucleosomes-free regions. This is consistent with previous biochemical studies indicating that repair of helix-distorting lesions by the NER pathway, including BPDE adducts, is inhibited in reconstituted nucleosomes relative to free DNA *in vitro* (17, 48). Inhibition of NER activity in nucleosomes is likely due to reduced accessibility of the DNA lesion to NER factors such as XPC, which plays a critical role in detecting DNA lesions and initiating NER. Consistent with this hypothesis, a prior study indicates that XPC binding to damaged DNA is significantly reduced when the lesion is packaged into nucleosome (17).

BPDE induces a variety of different guanine and adenine adducts, which differ in their structure, susceptibility to repair, and mutagenic mechanism (50). While our analysis indicates that BPDE adducts are formed preferentially in accessible linker DNA relative to nucleosomes, it is not clear whether the formation and repair of specific classes of BPDE adducts is affected differently by nucleosomes. Preliminary analysis of a small set of previously published mutations identified by single molecule mutation-sequencing (SMM-seq) in human cells following exposure to BPDE (12) indicated no substantial differences in the trinucleotide mutation patterns between nucleosomal DNA and non-nucleosomal (*e.g.* linker) regions (Fig. S13; Cosine similarity = 0.94). These findings suggest that similar mechanisms of BPDE mutagenesis are operating in both contexts, just at different rates.

Our analysis also indicates that other DNA-bound proteins, such as transcription factors, modulate BPDE adduct formation. Analysis of CTCF and SP1 binding sites, which were chosen because of their abundance in the genome and importance in regulating chromatin organization and transcription, revealed that damage formation was generally suppressed near the center of binding sites, where the transcription factor directly binds the DNA. These results are consistent with a recent report (12) and suggest a model where DNA-bound proteins may render the underlying DNA bases less accessible to react with BPDE. While repair is also inhibited by transcription factor binding, our results suggest that damage, not repair, correlates best with observed mutation patterns in the immediate vicinity (*i.e.*, 25 bp window) of TFBSs. Moreover, mutation patterns in the DNA surrounding TSSs, which are characterized by frequent SP1 binding sites and lower nucleosome occupancy, display a mutation peak that correlates with an increase in BPDE damage formation.

While a previous study observed that BPDE formation is elevated in active chromatin states, nucleosome-free DNase I hypersensitivity sites (DHS), and other accessible regions of the genome, they did not observe a link between elevated BPDE damage formation and mutagenesis in lung cancers (12). Instead, they concluded that repair inhibition in chromatin is the primary driver of mutation patterns in lung cancers. Importantly, we did observe that in regions with exceptionally high repair, such as the nucleosome-free regions surrounding CTCF binding sites, repair rates were more indicative of mutation patterns than damage, consistent with this previous report (12). However, our analysis of BPDE damage formation and repair in nucleosomes, which has not been analyzed previously, indicate that higher levels of adduct formation in more accessible linker DNA and minor-out positions in nucleosomes can explain elevated mutation rates in these chromatin contexts. We hypothesize that repair activity may not compensate for elevated damage formation in such chromatin contexts, because unlike DHS and other nucleosome-free regions, linker DNA and minor-out positions in nucleosomes may not have the same degree of accessibility to the NER machinery as they do to BPDE. For example, linker DNA regions are often bound by histone H1 (51), which may be less of a barrier to BPDE adduct formation than it is to NER. Future studies will be required to test this hypothesis.

It is important to note that the findings reported here have a few limitations which could be addressed in future studies. First, while our analysis utilizes high-quality nucleosome maps from human cells (14, 31, 32, 33, 35), these maps are not derived from lung cells (or lung cancers). Hence, it is possible that the patterns of damage formation and somatic mutations would be even more pronounced if a nucleosome map derived from lung cells was available. Moreover, it is possible that DNA lesions resulting from BPDE exposure could alter nucleosome positioning and/or stability in these cells (48, 49), which would also be intriguing to investigate in a future study. Second, while our analysis suggests a link between differential formations of BPDE adducts and lung cancer mutation rates in nucleosomes, TFBSs, and the regions surrounding TSSs, it will be important for future studies to further test these conclusions through experiments that deplete or alter these chromatin features. Third, although this study primarily focused on DNA lesions caused by BPDE, tobacco smoke contains many other compounds that are associated with lung cancer mutagenesis (52). It will be of interest in future studies to determine whether these carcinogens show similar patterns of damage formation in chromatin.

In summary, our analysis reveals the importance of altered damage formation in different chromatin contexts in shaping mutational heterogeneity in lung cancer genomes. By leveraging genome-wide maps of BPDE damage and repair activity, we show that BPDE damage formation is significantly modulated by nucleosomes, which can explain why somatic mutations in lung cancers are specifically elevated in linker DNA and minor-out positions in nucleosomes. We also describe a structural mechanism responsible for elevated BPDE formation at minor-out positions in nucleosomes. Taken together, these findings provide insight into how the packaging of DNA into nucleosome and the binding of transcription factors shapes the landscape of somatic mutations induced by BPDE and potentially other DNA damaging agents.

## Experimental procedures

### Parsing lung cancer mutation data

Lung cancer mutation data were obtained from The Cancer Genome Atlas (TCGA). Mutation calls from PCAWG were used for the majority of samples (38 lung adenocarcinomas [LUAD], and 48 lung squamous cell carcinomas [LUSC]) (29). An additional 57 LUSC samples were included, downloaded *via* GDC, excluding samples with cautionary notes, such as warnings of low purity or possible sample mix-up (30). These were filtered to include SNVs only, and for GDC obtained VCF files, only mutations with “PASS” in the filter column were included. When mutation calls were present both from PCAWG and GDC (4 samples), the PCAWG-based dataset was used, resulting in a total of 143 samples and 6,116,049 mutations. All mutations were transformed to hg19-based coordinates using the packages rtracklayer (53) and GenomicRanges (54) in R (55). Positions were transformed to 0-based indexing, and all mutations were made to reference the pyrimidine strand (*i.e.*, mutations with A or G on the reference strand were complemented for both the reference and alternative nucleotide, and the strand was set to ‘-’, whereas the remaining mutations were left as is and with strand set to ‘+’). All data was then pooled into a single file containing all mutations and sorted according to chromosome and position for downstream analyses.

### Calling BPDE lesion positions

Previously published BDPE damage-seq reads were obtained for cellular and naked DNA samples exposed to a 2 μM BPDE solution for 24 h (28) or cells exposed to a 25 μM BPDE solution for 2 h and naked DNA exposed to 3 μM BPDE for 2 h (12). Reads were aligned to the hg19 genome using Bowtie2 (56) v2.4.4 following adapter trimming with bbduk v38.95 from the BBtools suite (https://sourceforge.net/projects/bbmap/). Bowtie2 was run in paired-end mode with default parameters, and bbduk was run with the following parameters: ktrim = r k = 23 mink=16 hdist = 2 tpe tbo. Aligned reads were converted to bed format using SAMtools (57) and BEDtools (58). Concordant alignments were combined into a single read sequence and discordant alignments were kept only if they came from the first read in each pair (which is adjacent to the BPDE lesion). Duplicate read sequences within each replicate were removed, and then all replicates were combined for each experimental condition. BPDE lesions were called at the base immediately 5′ of each read sequence, and only lesions at guanine bases were retained for further analysis.

### Calling lesion positions in BPDE repair reads

Previously published BPDE translesion excision repair sequencing (tXR-seq) reads (11) were obtained for cells exposed to a 2 μM BPDE solution for 1 h. Reads were aligned to the hg19 genome using Bowtie2 (56) v2.4.4 following adapter trimming with bbduk v38.95 from the BBtools suite (https://jgi.doe.gov/data-and-tools/software-tools/bbtools/). Bowtie2 was run in single-end mode with default parameters, and bbduk was run with the following parameters: ktrim = r k = 23 mink=16 hdist = 2. Duplicate read sequences within each replicate were removed, and then all replicates were combined. Reads 22 to 29 bp long were retained for further analysis, as these read lengths were elevated above background lengths (Fig. S14*A*). Lesions were called at the position with the greatest guanine frequency for each read length (Fig. S14*B*).

### Generating nucleosome maps

The hybrid nucleosome map was produced from DNase (33) and MNase (32) maps as previously described (31). In brief, nucleosome dyads with precise rotational positioning were initially called using DNase-seq reads, and MNase-seq reads were then used to refine translational positioning. The MNase-only map was produced from MNase-seq (32) data using a pipeline designed for analysis of nucleosome periodicities (14). The pipeline was modified so that dyads were called throughout the human genome instead of only at mappable genic regions. Dyads were lifted over to the hg19 genome assembly using the University of California, Santa Cruz Genome Browser LiftOver tool (59).

### Excluding blacklisted regions

Prior to analysis, all nucleosome dyad centers and transcription factor binding sites within 1000 bp of any region in the ENCODE blacklist (60) were removed. TSSs from genes overlapping any part of the blacklist were also removed.

### Analyzing nucleosome periodicities

Nucleosome periodicities were analyzed using the mutperiod package (34). Mutation, damage, and repair data were counted in 2000-bp windows surrounding nucleosome dyads and normalized by trinucleotide context (for mutation and repair data) or a naked DNA control (for damage data). Period and signal to noise ratio (SNR) were calculated using a Lomb-Scargle periodogram, as previously described (14, 34). Nucleosome and linker regions were defined using the nucleosome repeat length of the related nucleosome map (also calculated with a Lomb-Scargle periodogram), and minor-in vs. minor-out regions were derived from previously published structural models (36). The exact positions encompassed by the minor-in and minor-out regions, including the transitionary “minor-in to minor-out” and “minor-out to minor-in” positions can be reproduced using the mutperiod (34) script at https://github.com/bmorledge-hampton19/mutperiod/blob/master/python_packages/mutperiod/mutperiodpy/GeneratePlotnineFigures.py.

## Analysis of solvent accessible surface area in nucleosomes structures

We used the dr-sasa (61) software with default parameters to calculate the solvent accessible surface area ) of the N2 position of guanine bases for the human (2CV5), *Xenopus* (1KX5), and *Xenopus* with 601 DNA sequence (3LZ0) nucleosome structures (62, 63, 64). Statistical analysis was performed using the Mann-Whitney test in the GraphPad Prism (https://www.graphpad.com/) software.

### Calling TFBS midpoints

CTCF and SP1 binding sites were originally called by Khurana *et al.* (41) using data from the ENCODE project (40). From these data, we retrieved all binding sites classified as “known”. Since each set of binding sites was composed of multiple motifs without clear consensus on midpoints, offsets had to be manually calculated using the expected motif sequence and applied to each motif to reduce binding sites to a standardized, single-nucleotide position. For binding motifs with a midpoint at a half-base position, the position was rounded up if the binding site was on the plus strand and rounded down if it was on the minus strand. (See data availability section for formatted midpoints.)

### Calling and stratifying TSSs

TSSs were called using the GENCODE hg19 annotations (44). Specifically, only genes described as “protein_coding” were retained, and entries from mitochondrial chromosomes were ignored. After filtering these genes using the ENCODE blacklist, TSSs were called at the 5′ end of each gene. TSSs were stratified by expression data from the A549 cell line (65) for damage and mutation data and expression data from the GM12878 cell line (66) for repair data. In brief, we retrieved the gene IDs associated with the upper and lower quartiles of each of these expression data sets and used these to stratify the TSSs into high and low expression data sets.

### Analyzing data relative to TFBSs and TSSs

Mutperiod (34) was used to count mutation, damage, and repair positions relative to TFBSs and TSSs, similar to the analysis of nucleosome periodicities (*i.e.*, mutation, damage, and repair data were counted in 2000-bp windows centered on these features and normalized by trinucleotide context [for mutation and repair data] or a naked DNA control [for damage data]). This is possible because TFBS midpoints and TSSs are single-nucleotide features, just like nucleosome dyad centers, so mutperiod functions identically on them. No periodicity analysis was performed relative to these features, but in cases where a nucleosome pattern was present, nucleosomal and linker regions were defined by calling peaks of dyad counts relative to the features.

### Correlating damage or repair with mutagenesis

Pearson correlation coefficients and related *p*-values were obtained using the pearsonr function from scipy (67) with the alternative hypothesis set to “two-sided”.

### Sequence logo generation

The CTCF sequence logo was generated using WebLogo 3 (68) with an equiprobable background composition. Input sequences were obtained by retrieving the 50 bp (25 bp on either side) flanking each analyzed CTCF midpoint.

### Characterizing mutation signatures

Mutation signatures in nucleosomal *versus* non-nucleosomal (*e.g.*, linker) DNA were characterized using BPDE-induced mutations from previously published SMM-seq data (12). These mutation data were first lifted over to the hg19 genome using the University of California, Santa Cruz Genome Browser LiftOver tool (59). Then, each mutation was categorized as either nucleosomal, if it was within 73 bp of one of the nucleosome dyads described in the MNase-only nucleosome map (32), or linker, if it was encompassed by none of these regions. The mutation signatures were then created from the trinucleotide contexts of these two mutation cohorts and compared by cosine similarity.

### Code availability.

The code developed for this analysis, along with instructions on how to implement it to reproduce results, can be found at the following GitHub repository: https://github.com/bmorledge-hampton19/bpde_chromatin_analysis.

## Data availability

Controlled-access TCGA mutation data from PCAWG (29) were downloaded from the AWS S3 bucket s3://pcawg-tcga/consensus_snv_indel/final_consensus_passonly.snv_mnv_indel.tcga.controlled.maf.gz at the https://bionimbus-objstore-cs.opensciencedatacloud.org endpoint with a PDC access token. Remaining TCGA data were downloaded from https://portal.gdc.cancer.gov (30), only including WGS-based VCF-files.

The BPDE-induced mutations used to analyze mutation signatures in nucleosomal *versus* linker regions were derived from previously published SMM-seq data (12). The raw data used to derive these mutations can be accessed through the NCBI Sequence Read Archive under the project ID PRJNA1179438 (https://www.ncbi.nlm.nih.gov/sra/?term=PRJNA1179438).

The 24h BPDE damage map can be accessed through the Gene Expression Omnibus (GEO) under the accession ID GSE224001 (https://www.ncbi.nlm.nih.gov/geo/query/acc.cgi?acc=GSE224001) (28).

The 2h BPDE Damage map can be accessed through the NCBI Sequence Read Archive under project ID PRJNA1179438 (https://www.ncbi.nlm.nih.gov/sra/?term=PRJNA1179438) (12).

The BPDE tXR-seq map used to call repair positions can be accessed through GEO under the accession ID GSE97675 (https://www.ncbi.nlm.nih.gov/geo/query/acc.cgi?acc=GSE97675) (11).

The MNase and DNase maps used to produce the hybrid and MNase-only nucleosome maps can be accessed through GEO under the accession IDs GSE36979 (https://www.ncbi.nlm.nih.gov/geo/query/acc.cgi?acc=GSE36979) (32) and GSE31388 (https://www.ncbi.nlm.nih.gov/geo/query/acc.cgi?acc=GSE31388) (33), respectively.

The ENCODE blacklist (60) can be downloaded from https://github.com/Boyle-Lab/Blacklist/blob/master/lists/hg19-blacklist.v2.bed.gz.

The TFBSs (40, 41) used in this analysis can be found in the “maintained_data” directory at the root of this research’s code repository: https://github.com/bmorledge-hampton19/bpde_chromatin_analysis/maintained_data.

The hg19 genome annotations used to derive TSSs can be found in the GENCODE database (44) at https://www.gencodegenes.org/human/release_19.html. The expression data used to stratify these TSSs for A549 (65) and GM12878 (66) cell lines can be found at https://depmap.org/portal/cell_line/ACH-000681?tab=overview and https://egg2.wustl.edu/roadmap/web_portal/processed_data.html#RNAseq_uni_proc respectively. The formatted TSS sites used in this analysis can be found at https://github.com/bmorledge-hampton19/bpde_chromatin_analysis/tree/main/maintained_data/TSSs.

## Supporting information

This article contains supporting information.

## Conflict of interest

The authors declare that they have no conflicts of interest with the contents of this article.
