# Supplementary material for: Altered benzo[a]pyrene adduct formation in nucleosomes establishes distinct mutational patterns in lung cancer
Source: J Biol Chem. 2026 Feb 16;302(4):111291. doi: 10.1016/j.jbc.2026.111291 (PMC13010920; doi:10.1016/j.jbc.2026.111291)
Supplement: Supplemental Tables and Figures [file mmc1.docx]

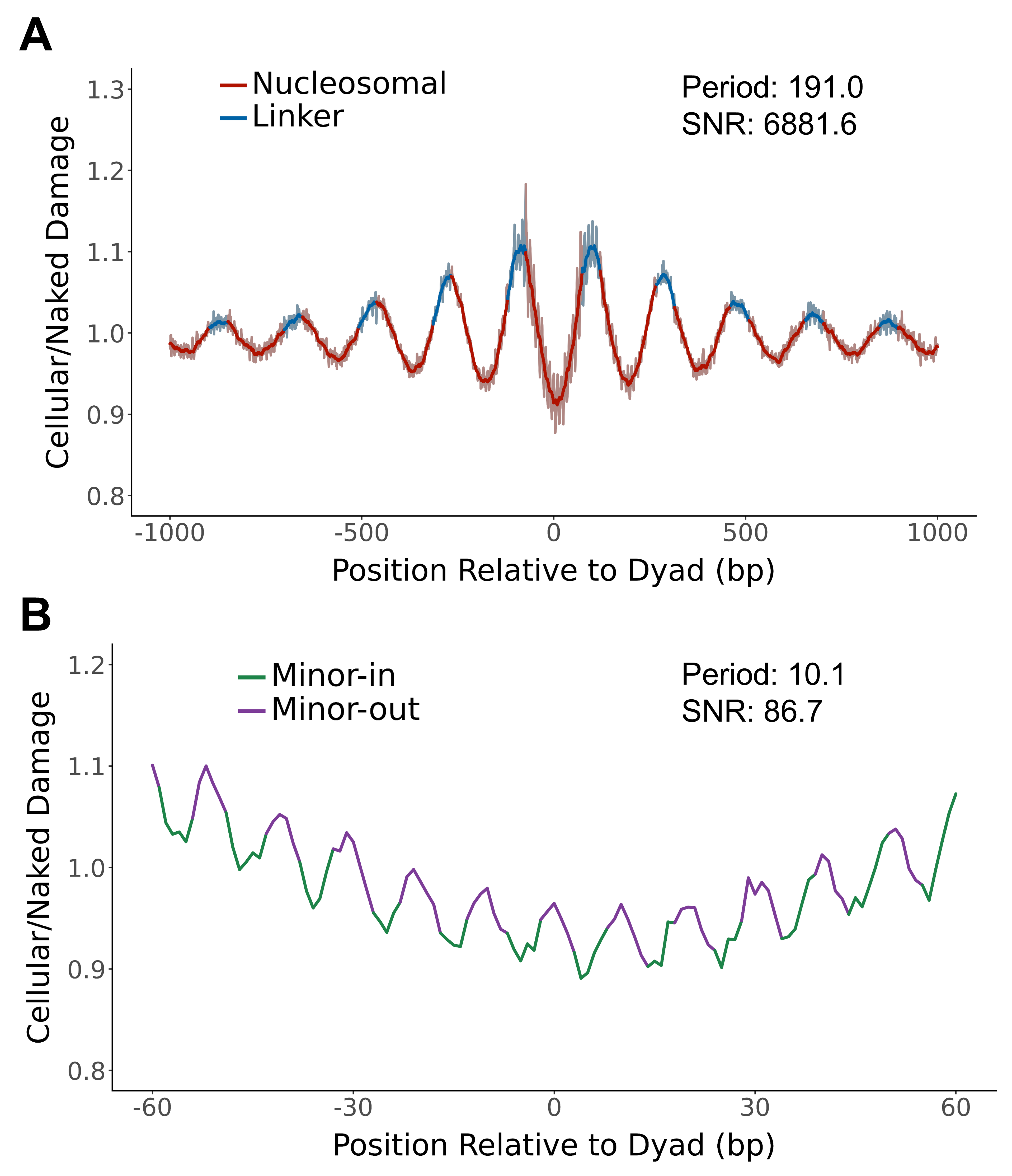


**Figure S1: BPDE damage patterns in nucleosomes are consistent across different exposure conditions.** (**A,B**) BPDE damage enrichment (2h exposure) with respect to translational (A) and rotational (B) periodicity. Data are normalized using a naked DNA control. Dyad-relative positions are oriented 5’ to 3’ relative to the plus strand and are combined over both strands. For translational data (A) darker lines represent data smoothed in a sliding 11-bp window and are superimposed on the lighter-colored raw data. Rotational data (B) is not smoothed.


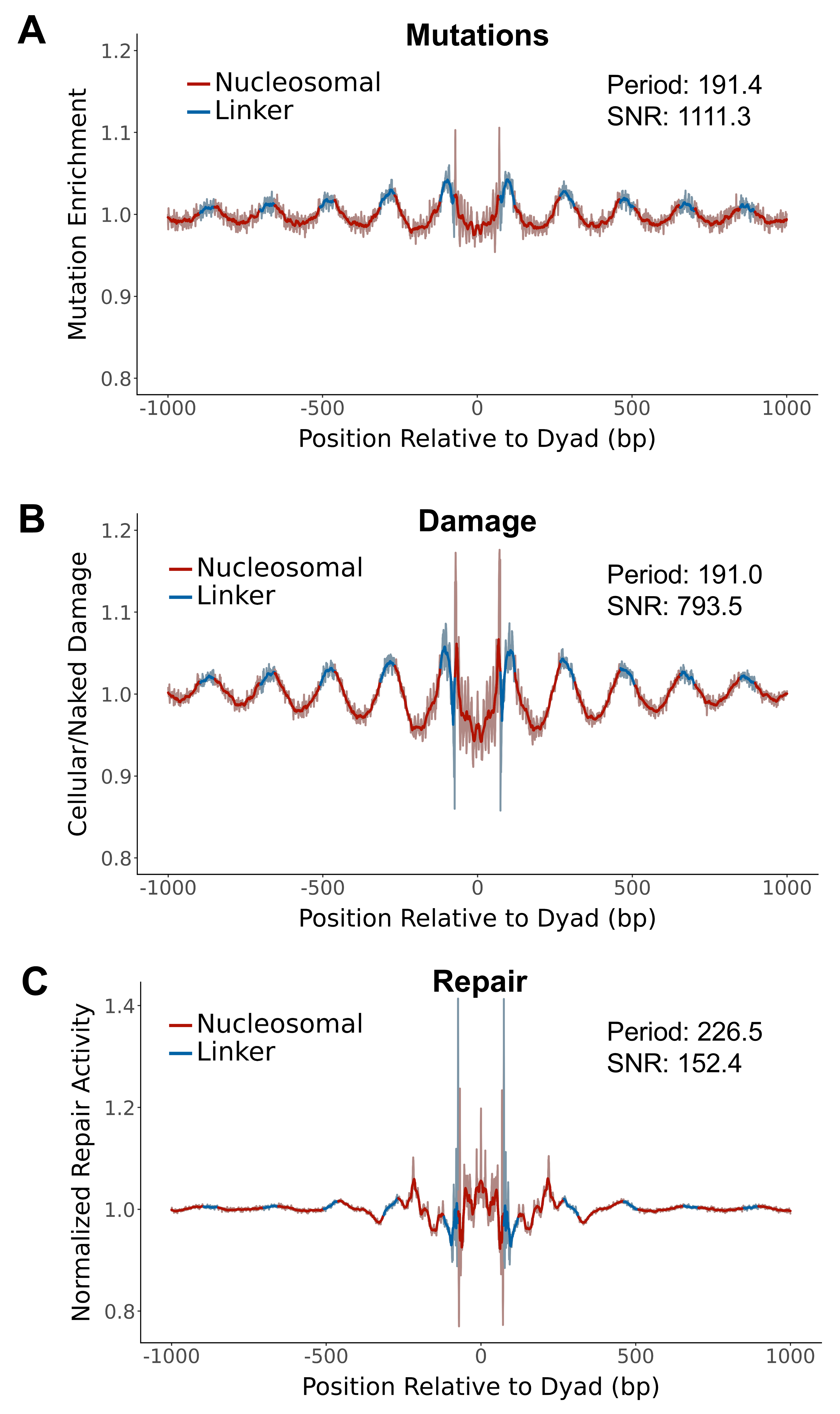


**Figure S2: Translational periodicities for lung cancer mutagenesis and BPDE damage are also present in an MNase-only nucleosome map.** (**A-C**) Lung cancer mutation enrichment (A), BPDE damage enrichment (24h exposure) (B), and BPDE repair activity (C) with respect to translational nucleosome positioning based on an MNase-only nucleosome map. Mutation data (A) and repair data (C) are normalized by trinucleotide context. Damage data (B) are normalized by a naked DNA control. Dyad-relative positions are oriented 5’ to 3’ relative to the plus strand and are combined over both strands. Darker lines represent data smoothed in a sliding 11-bp window and are superimposed on the lighter-colored raw data.


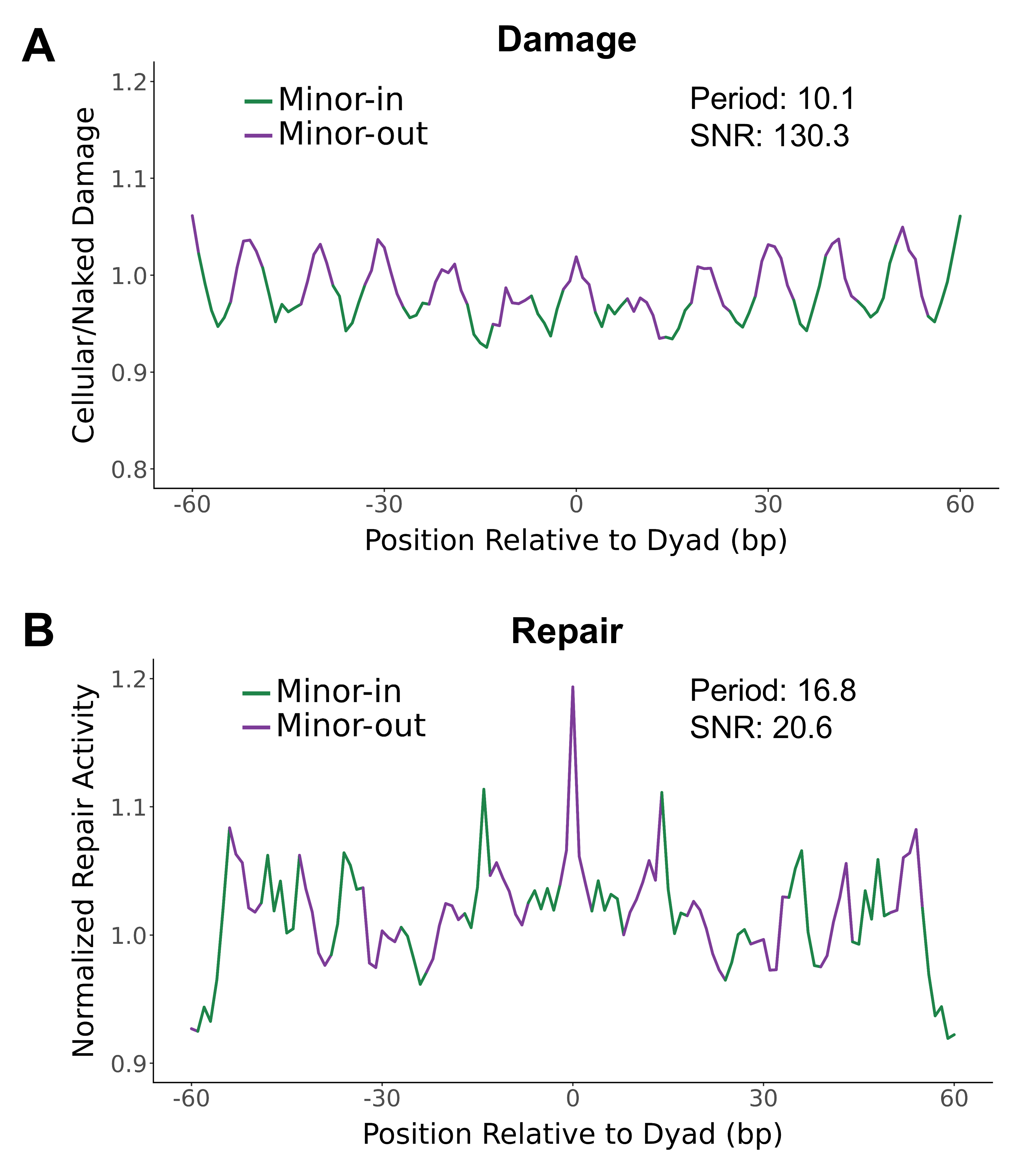


**Figure S3: The rotational periodicity for BPDE damage is also present in an MNase-only nucleosome map.** (**A,B**) BPDE damage enrichment (24h exposure) (A) and repair activity (B) with respect to rotational nucleosome positioning based on an MNase-only nucleosome map. Damage data (A) are normalized by a naked DNA control. Repair data are normalized by trinucleotide context. Dyad-relative positions are oriented 5’ to 3’ relative to the plus strand and are combined over both strands.


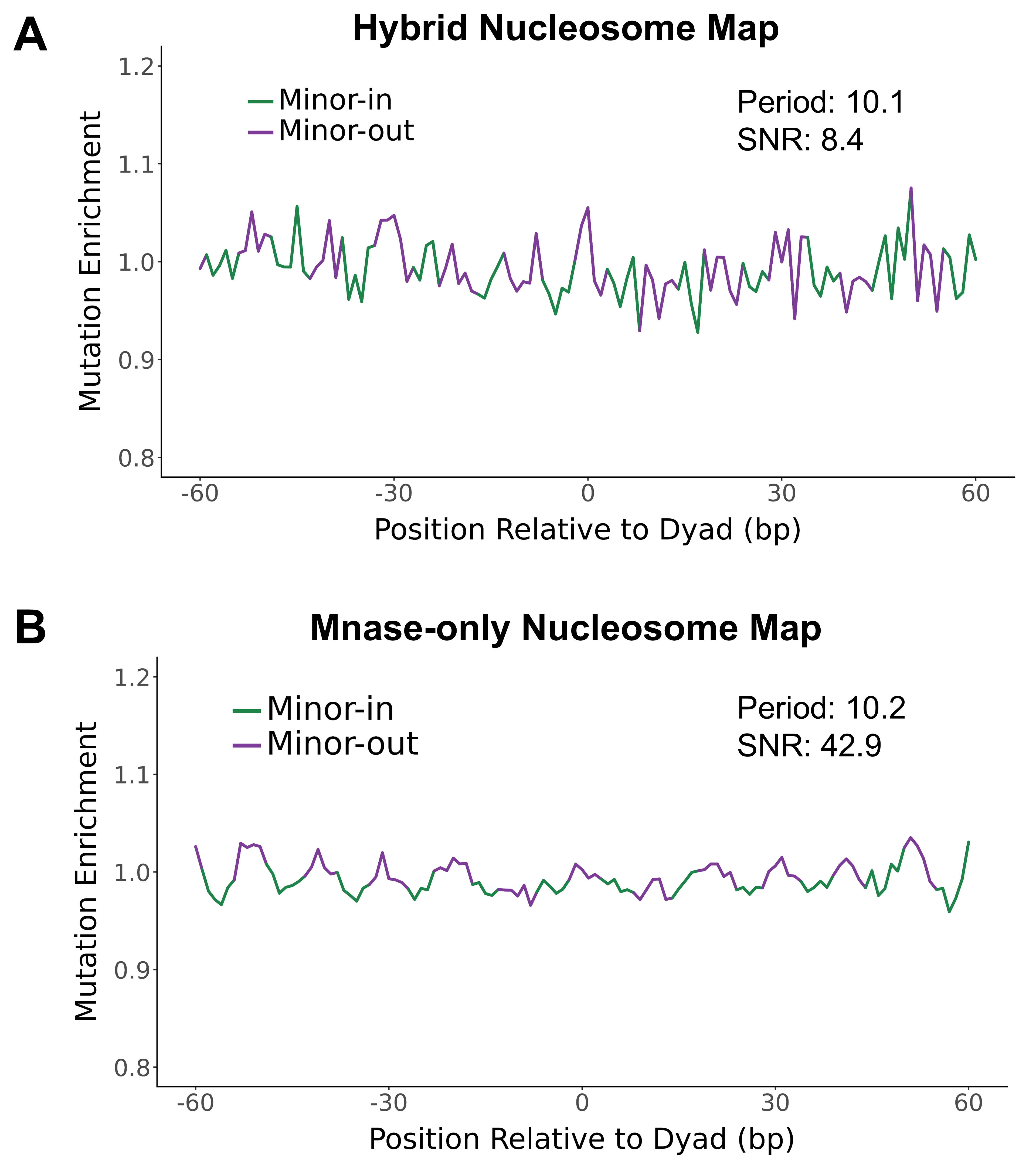


**Figure S4: Lung cancer mutations are slightly enriched at minor-out positions for an MNase-only nucleosome map.** (**A,B**) Lung cancer mutation enrichment with respect to rotational nucleosome positioning based on a hybrid DNase-MNase (A) or MNase-only (B) nucleosome map. Data are normalized to trinucleotide context. Dyad-relative positions are oriented 5’ to 3’ relative to the plus strand and are combined over both strands.


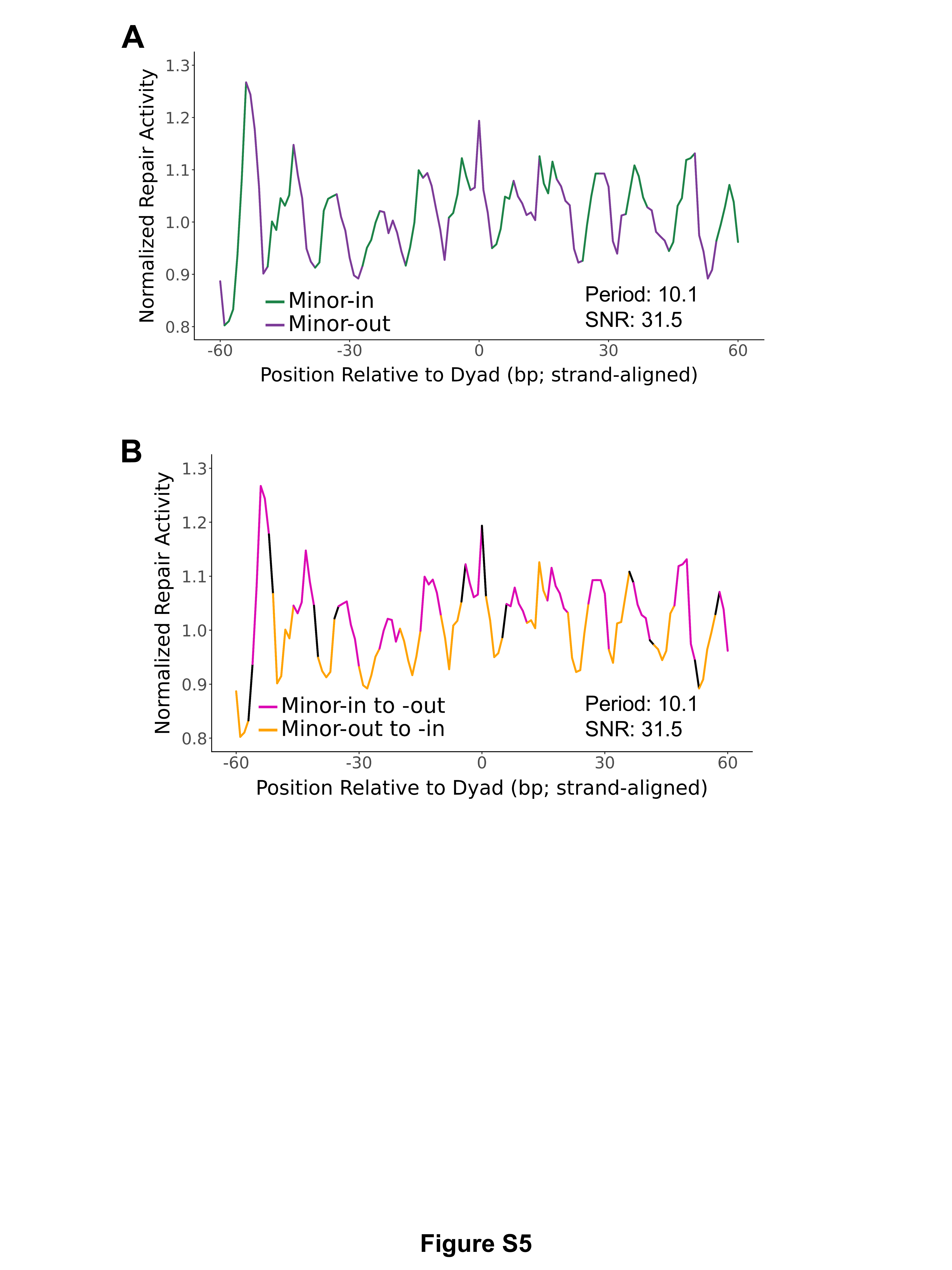


**Figure S5: Strand-specific BPDE repair patterns are also present in an MNase-only nucleosome map.** (**A,B**) BPDE repair activity with respect to rotational nucleosome positioning based on an MNase-only nucleosome map, highlighting either the position of the minor groove (A) or transitions between these positions (B). Data are normalized to trinucleotide context. Dyad-relative positions are oriented 5’ to 3’ for both strands. (I.e., minus-strand positions were inverted about the dyad axis so that both strands are aligned in the same direction.)


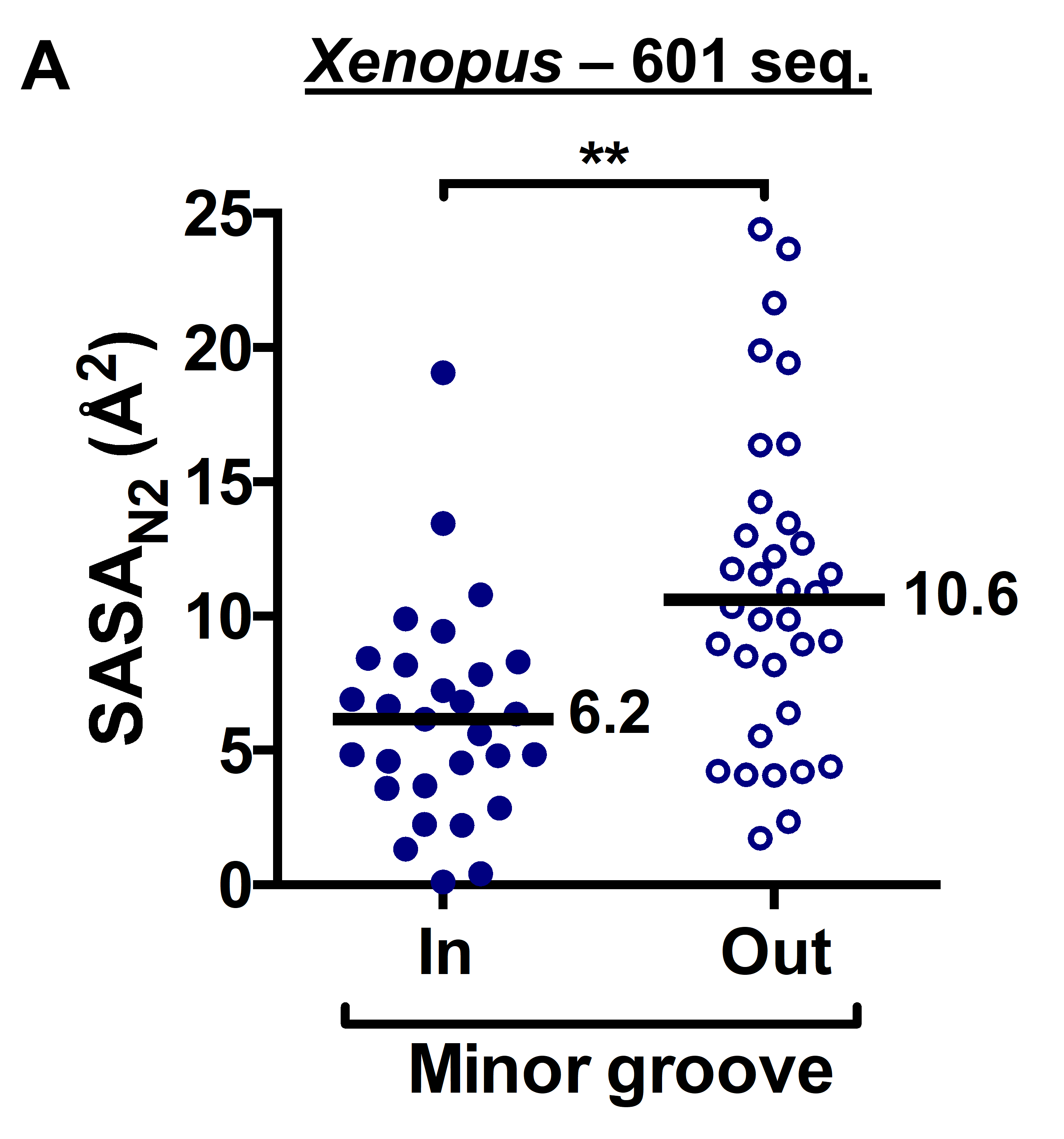


**Figure S6: Structural analysis indicates that the solvent accessibility of the reactive N2 position of guanine is modulated by rotational positioning in nucleosomes.** (**A**) Solvent accessible surface area of the N2 atom in guanine nucleotides (SASA_N2_) for minor-in and minor-out positions in the *Xenopus* nucleosome structure containing the 601 nucleosome positioning sequence (3LZ0). The median SASA_N2_ for minor-in and minor-out positions are indicated. **P < 0.001 based on Mann-Whitney test.


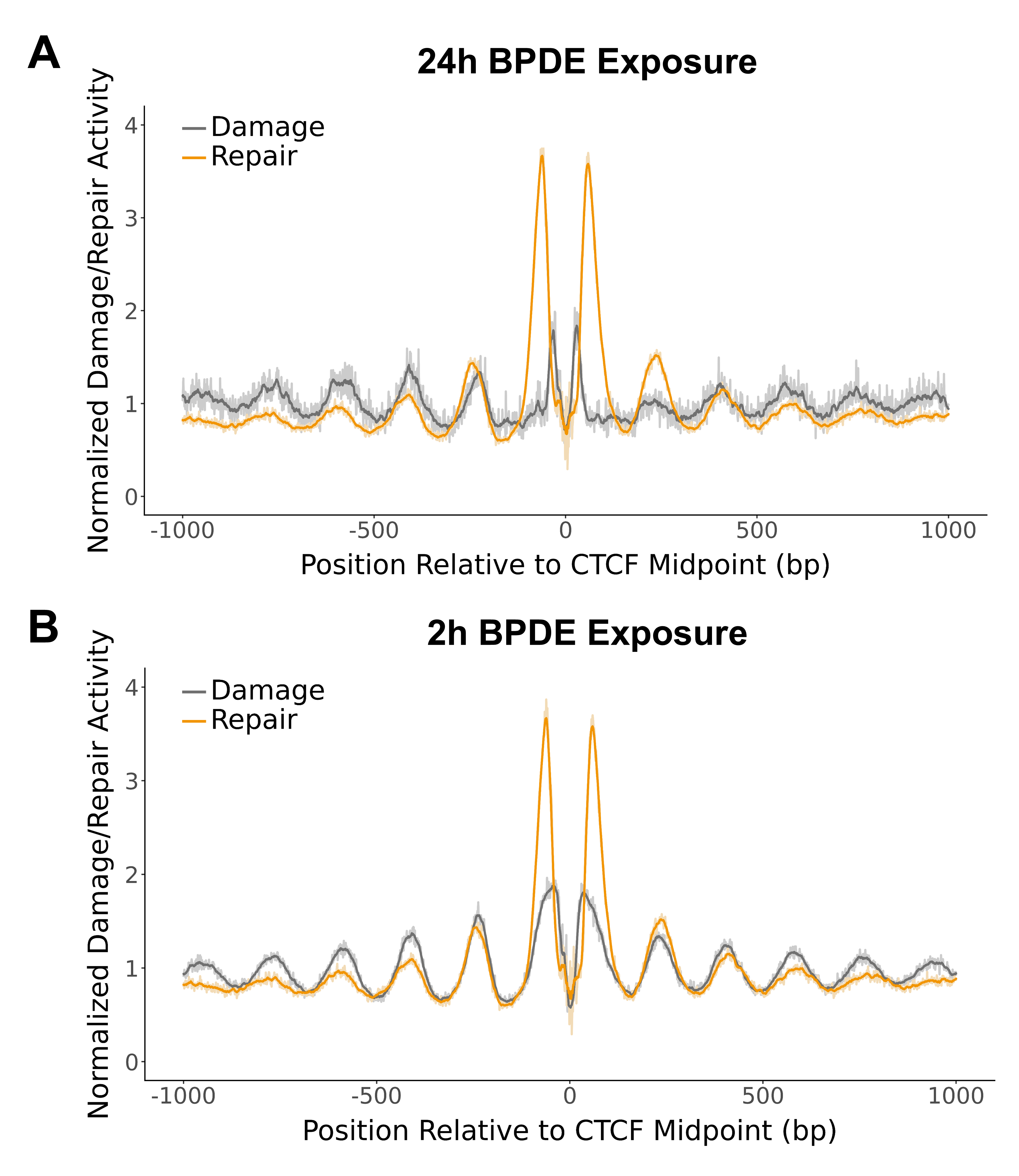


**Figure S7: BPDE damage patterns around CTCF binding sites are modulated by exposure conditions.** (**A,B**) Damage enrichment surrounding CTCF binding sites after 24h (A) or 2h (B) of BPDE exposure. Repair data are identical across the two panels and were collected after 1h of BPDE exposure. Damage data are normalized to a naked DNA control, while repair data are normalized by trinucleotide context. Positions are oriented 5’ to 3’ relative to the strand containing the CTCF binding motif and are combined over both strands. Darker lines represent data smoothed in a sliding 11-bp window and are superimposed on the lighter-colored raw data.


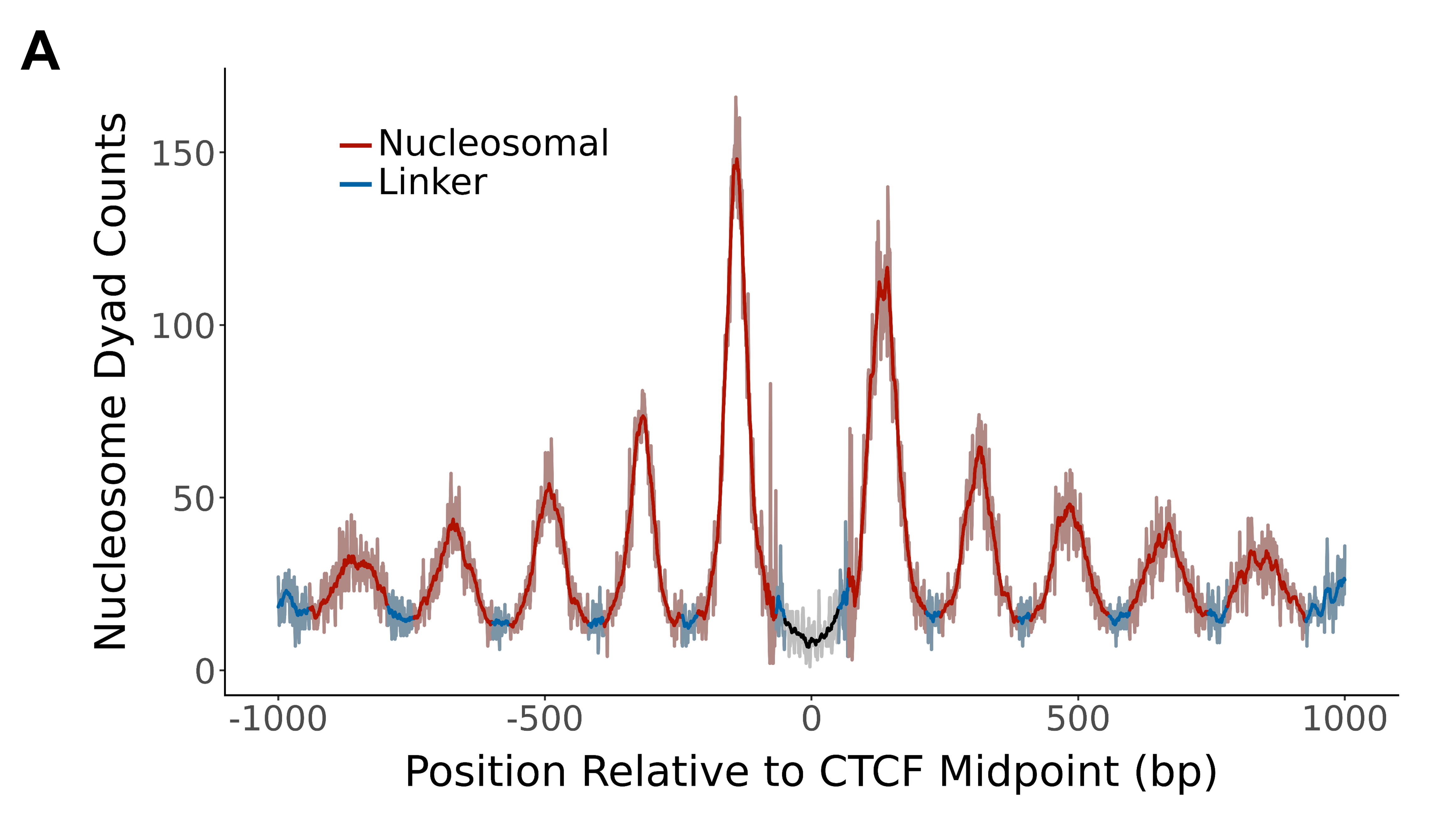


**Figure S8: Nucleosomes flanking CTCF binding sites display consistent positioning.** (**A**) Counts of nucleosome dyad positions from a hybrid DNase-MNase nucleosome map relative to CTCF binding sites. Nucleosomal and linker regions were determined by calling peaks of nucleosome dyad positions, omitting regions within 100 bp of the CTCF binding sites. Darker lines represent data smoothed in a sliding 11-bp window and are superimposed on the lighter-colored raw data.


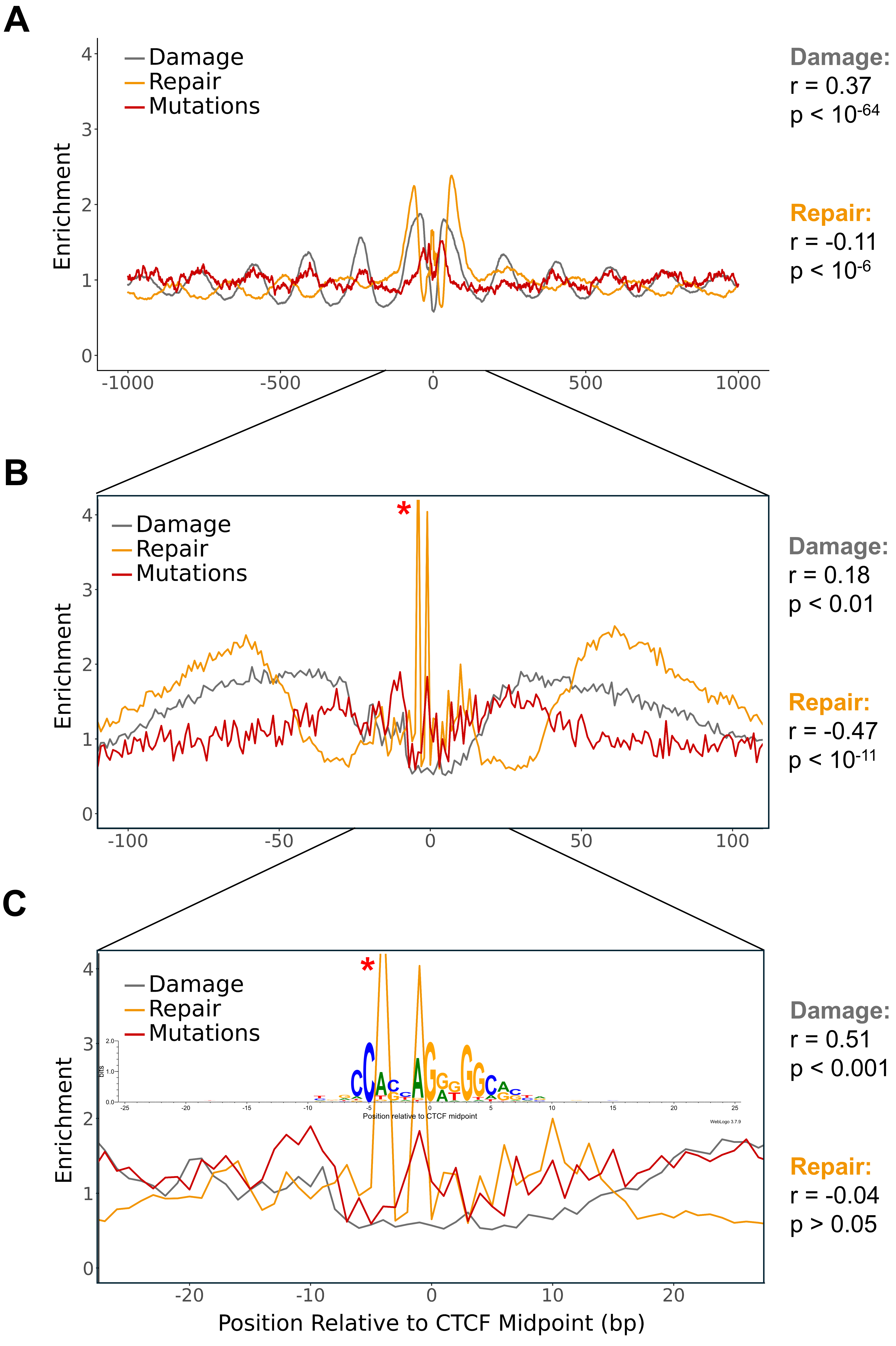


**Figure S9: BPDE repair activity normalized by damage negatively correlates with mutagenesis in regions flanking CTCF binding sites.** (**A-C**) BPDE damage enrichment (2h exposure), BPDE repair activity, and lung cancer mutation enrichment surrounding CTCF binding sites in a 2000-bp window (A), 200-bp window (B), or 50-bp window (C). For the 2000-bp window (A), data are smoothed in a sliding 11-bp window. For the 200-bp window (B) and 50-bp window (C), data are not smoothed. Damage data are normalized by a naked DNA control, repair data are normalized using the non-normalized damage data, and mutation data are normalized by trinucleotide context. The red asterisks indicate a position of particularly high normalized repair activity which was cut off to increase readability for the rest of plot (enrichment = 5.11). Positions are oriented 5’ to 3’ relative to the strand containing the CTCF binding motif and are combined over both strands. The sequence logo (C) represents the relative information content of positions surrounding CTCF binding midpoints.


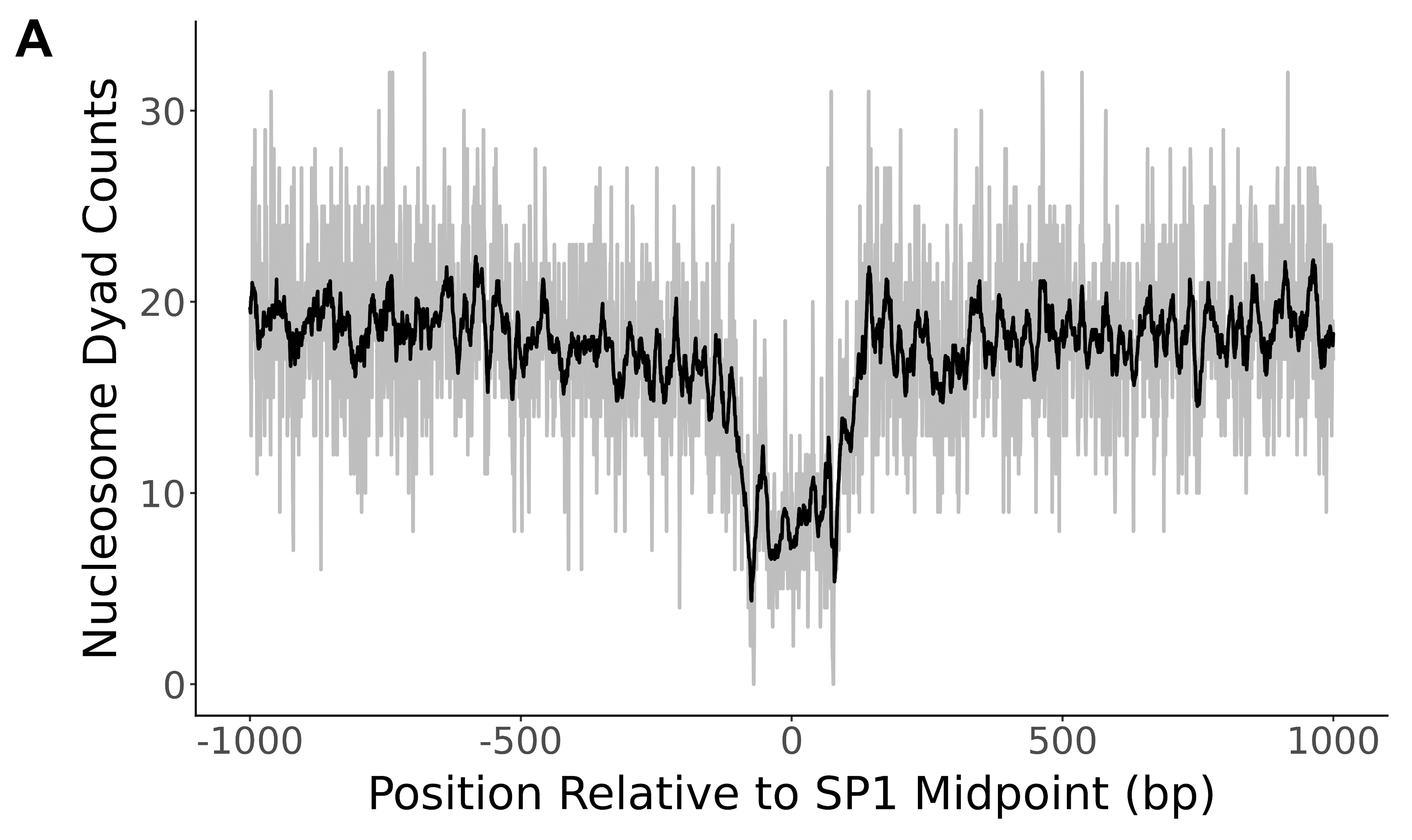


**Figure S10: Nucleosome positions do not show a consistent pattern around SP1 binding sites.** (**A**) Counts of nucleosome dyad positions from a hybrid DNase-MNase nucleosome map relative to SP1 binding sites. Darker lines represent data smoothed in a sliding 11-bp window and are superimposed on the lighter-colored raw data.


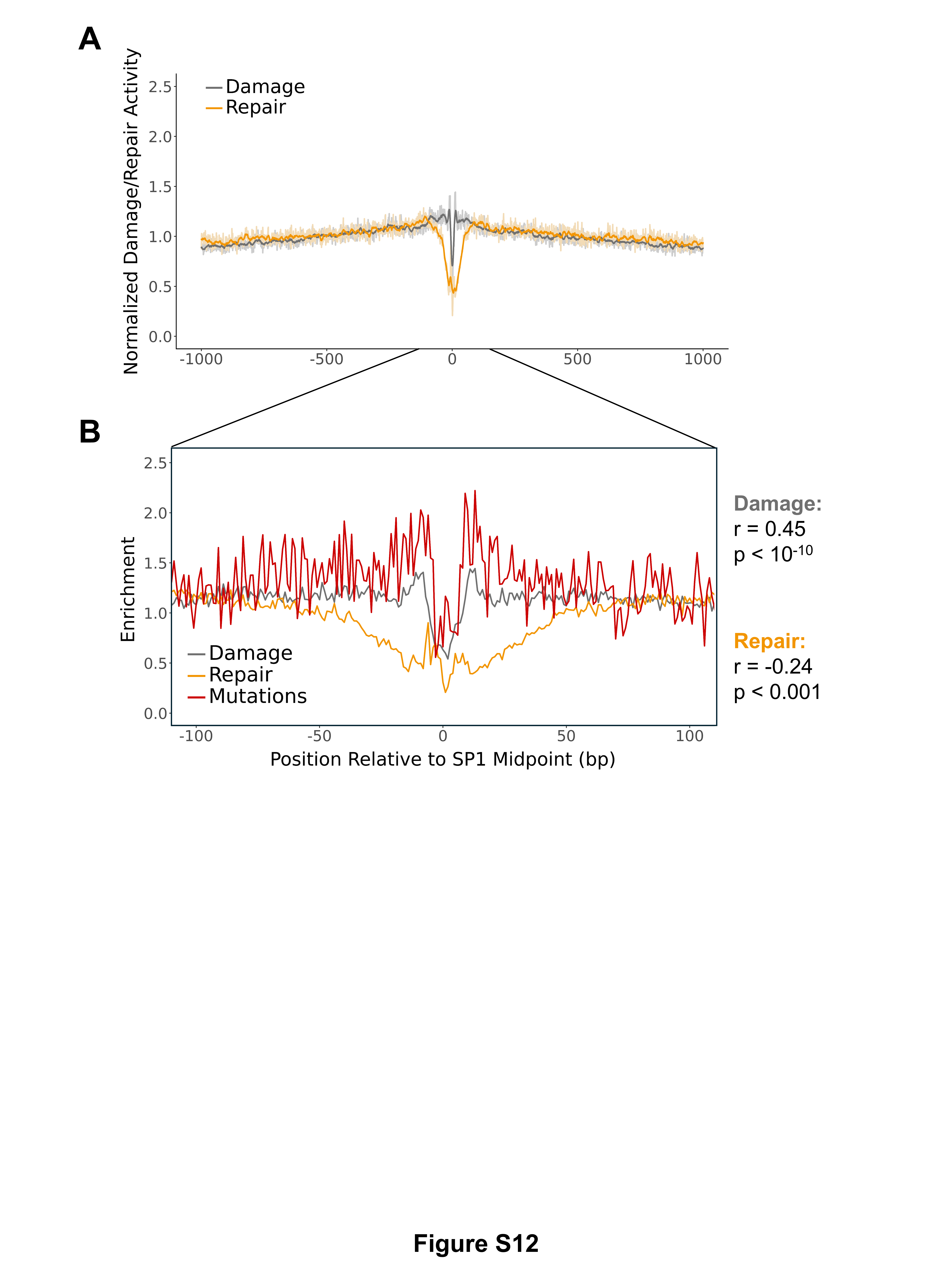


**Figure S11: BPDE repair activity normalized by damage negatively correlates with mutagenesis in regions flanking SP1 binding sites.** (**A,B**) BPDE damage enrichment (2h exposure), BPDE repair activity, and lung cancer mutation enrichment surrounding SP1 binding sites in a 2000-bp window (A) or 200-bp window (B). For the 2000-bp window (A), data are smoothed in a sliding 11-bp window and superimposed on the lighter, raw data. For the 200-bp window (B), data are not smoothed. Damage data are normalized by a naked DNA control, while repair and mutation data are normalized by trinucleotide context. Positions are oriented 5’ to 3’ relative to the strand containing the SP1 binding motif and are combined over both strands.

**
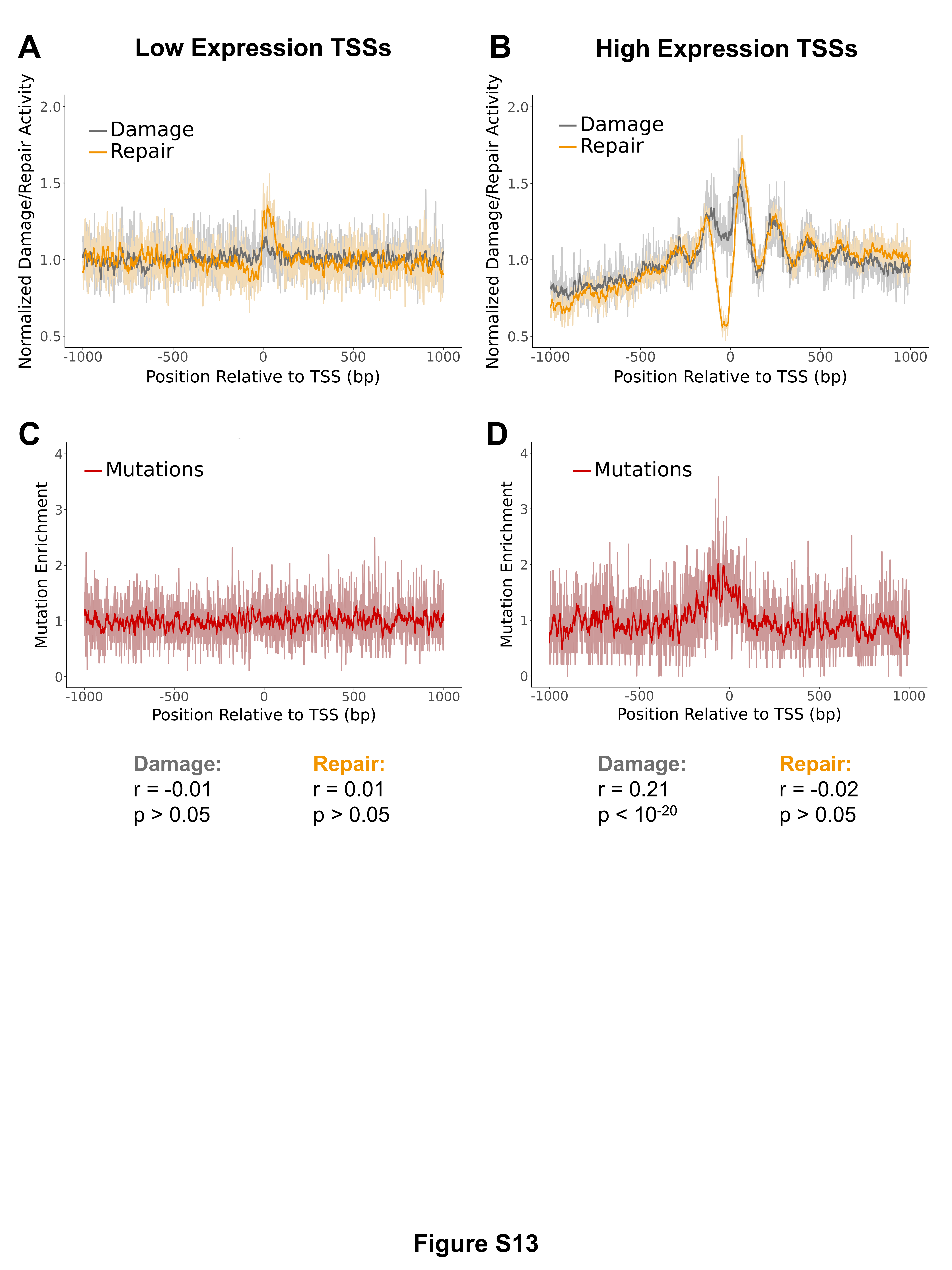
**

**Figure S12: The correlation between BPDE damage enrichment and lung cancer mutagenesis is linked to transcriptional activity.** (**A-D**) BPDE damage enrichment (2h exposure) vs. repair activity (A,B) and lung cancer mutation enrichment (C,D) surrounding transcription start sites (TSS). TSSs are stratified by gene expression to highlight the lower (A,C), and upper (B,D) quartiles. Damage data are normalized by a naked DNA control, while repair and mutation data are normalized by trinucleotide context. Positions are oriented 5’ to 3’ relative to the transcribed strand and are combined over both strands. Darker lines represent data smoothed in a sliding 11-bp window and are superimposed on the lighter-colored raw data.


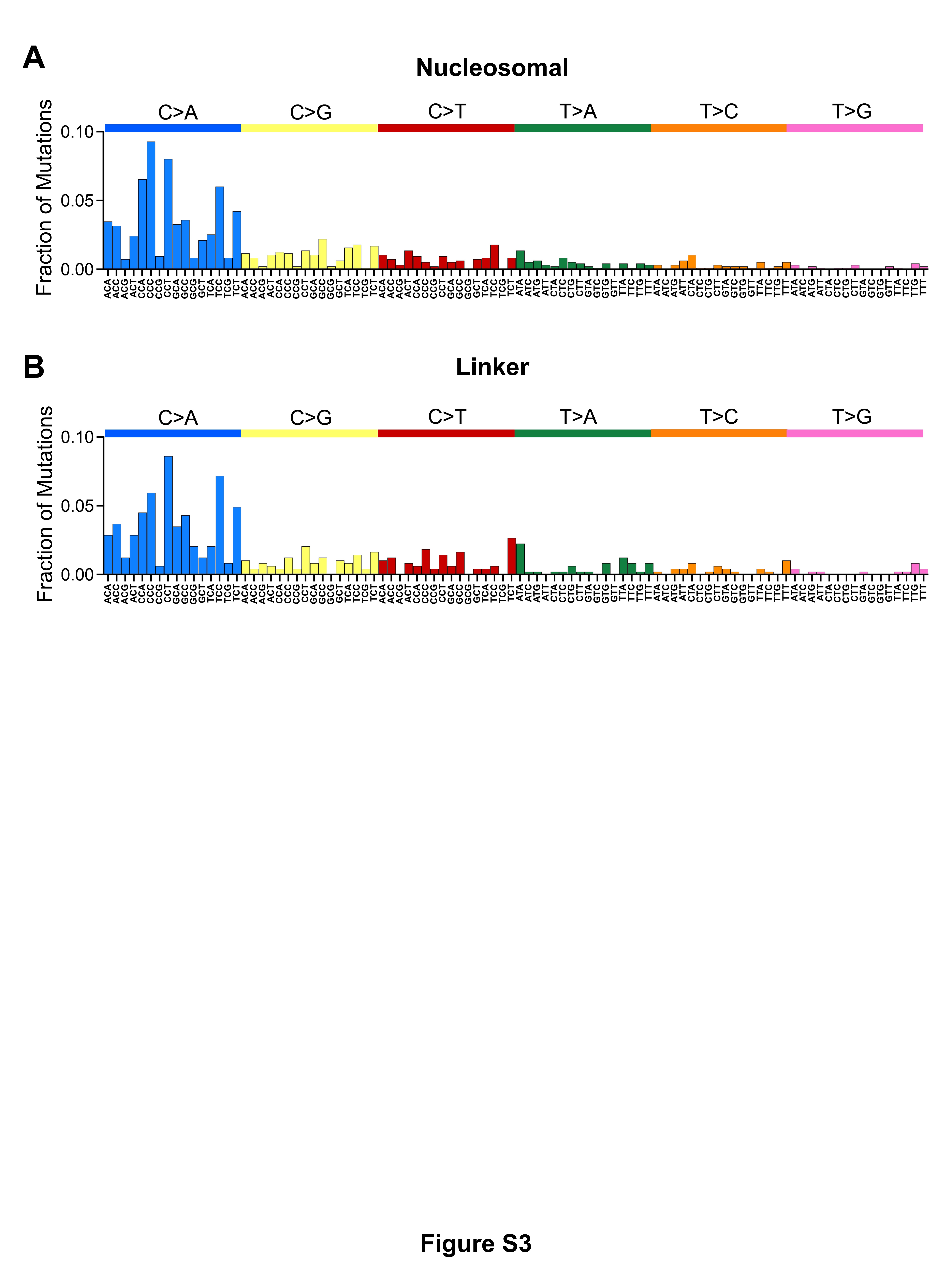


**Figure S13: Signatures of BPDE mutagenesis are similar across nucleosomal and linker positions.** (**A,B**) Trinucleotide mutation signatures for BPDE-induced mutagenesis in nucleosomal (A) and non-nucleosomal (e.g. linker) (B) DNA. Mutation fractions were calculated independently for each plot.


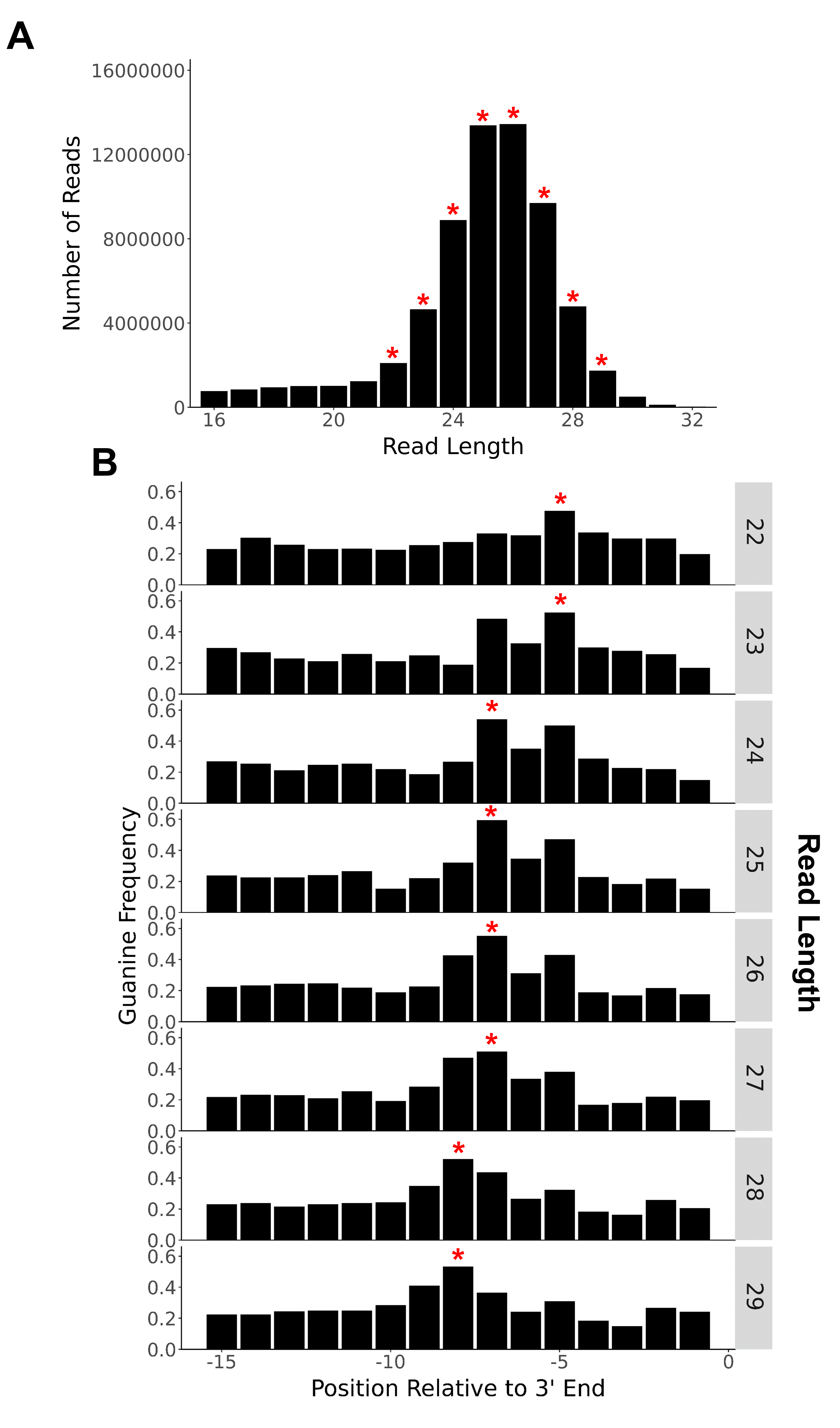


**Figure S14: Lesion calling in tXR-seq reads is decided by the most common read lengths and guanine frequencies relative to the 3’ end.** (**A**) Counts of tXR-seq read lengths. (**B**) Guanine frequencies relative to the 3’ ends of tXR-seq reads, stratified by read length. Red asterisks denote read lengths elevated above background (A) and positions with the highest guanine frequency for each read length (B). These read lengths and positions were used to call BPDE lesions in tXR-seq reads.
